# Primary and secondary cardiovascular disease prevention interventions targeting lifestyle risk factors in women: A systematic review and meta-analysis

**DOI:** 10.3389/fcvm.2022.1010528

**Published:** 2022-11-09

**Authors:** Kaylee Slater, Kim Colyvas, Rachael Taylor, Clare E. Collins, Melinda Hutchesson

**Affiliations:** ^1^School of Health Sciences, College of Health, Medicine and Wellbeing, University of Newcastle, Callaghan, NSW, Australia; ^2^Food and Nutrition Research Program, Hunter Medical Research Institute, New Lambton Heights, NSW, Australia

**Keywords:** cardiovascular disease, women, lifestyle, behavior change, randomized control trial (RCT)

## Abstract

**Background and aims:**

Over seven million women die from cardiovascular disease (CVD) annually. While lifestyle modification is recommended for CVD prevention, there are no systematic reviews evaluating the effectiveness of interventions targeted to women. The primary aim of this systematic review is to determine the efficacy of primary and secondary CVD prevention interventions targeting lifestyle risk factors in women.

**Methods:**

Six electronic databases were searched up to January 2022. Eligible studies included randomized controlled trials of primary or secondary CVD prevention interventions targeting CVD lifestyle risk factors (diet, physical activity, sedentary behavior, smoking, alcohol, sleep, and weight management) in women (≥18 years) that reported CVD risk markers or lifestyle risk factors. Meta-analyses were conducted on CVD risk markers and body mass index (BMI), and the level of evidence was applied to the GRADE (Grading of Recommendations Assessment, Development and Evaluation) criteria and reported.

**Results:**

Thirty-five RCTs were included (24 primary and 11 secondary prevention). Meta-analyses demonstrated that lifestyle CVD prevention interventions achieved statistically significant reductions in BMI at ≤ 6 months (0.95 kg/m^2^, 95% CI = 0.54 to 1.35, *p* < 0.0001), 12 months (0.61 kg/m^2^, 95% CI = 0.07 to 1.16, *p* = 0.03) and >12 months (0.58 kg/m^2^, 95% CI = 0.01 to 1.16, *p* = 0.05), and systolic blood pressure (mmHg) at ≤ 6 months (3.51, *p* < 0.001).

**Conclusions:**

Lifestyle interventions are important for the prevention of CVD in women, specifically to reduce systolic blood pressure in the short term (≤ 6 months) and BMI long term (>12 months).

**Systematic review registration:**

https://osf.io/bkwqm, identifier: osf-registrations-bkwqm-v1.

## Introduction

Worldwide, over seven million women die from cardiovascular disease (CVD) annually (1). Notwithstanding global declines in CVD mortality over the past three decades, data between 1979 and 2011 highlights little improvement in CVD incidence or mortality among women < 55 years, where there was a 0.1% decrease in annual CVD mortality from 2000 to 2011 (2, 3).

Risk factors associated with CVD include advancing age, hypertension, dyslipidaemia, obesity, diabetes, smoking, sedentary behavior, poor diet quality and a family history of premature CVD (2). Women may also be affected by sex-specific CVD risk factors including hypertensive disorders of pregnancy (HDP), premature delivery, gestational diabetes, early or surgical menopause, early menarche, and breast cancer treatment (2, 4). The current American Heart Association evidence-based guidelines for CVD prevention in women acknowledged these sex-specific CVD risk factors, expanding the sex-specific recommendations (5). They also reiterate the significance of lifestyle modifications, including smoking cessation, maintaining a healthy body weight, improving diet quality, increasing physical activity, and reducing alcohol consumption for primary and secondary prevention of CVD in women (6–8).

Compared with men however, it is understood that women are less likely to be diagnosed appropriately and efficiently, or receive CVD preventive care (9). To improve CVD care for women, it is important that we observe sex-specific interventions to ensure advice is tailored and appropriate. Systematic reviews of randomized controlled trials (RCTs), involving both men and women have shown promising results using dietary intake, physical activity, digital health, and community programs for primary and secondary prevention of CVD (10–13). However, women with sex-specific risk factors would benefit from interventions targeted to them and their long-term health risks.

There is an opportunity to utilize existing experimental evidence evaluating CVD prevention interventions for women to guide further research and implementation in this field. To our knowledge there has been no recent systematic review or meta-analysis evaluating the efficacy of CVD prevention interventions targeting lifestyle risk factors for women. Therefore, the aim of this systematic review and meta-analysis was to evaluate the efficacy of primary and secondary CVD prevention interventions targeting lifestyle risk factors (dietary intake, physical activity, sedentary behavior, alcohol intake, sleep quality, smoking and weight management) in adult women. As a secondary aim, the association between length of intervention (≤ 6 months, 12 months, and ≥12 months) and other fixed-effect moderators including level of prevention (primary or secondary prevention), type of lifestyle intervention and number of lifestyle risk factors targeted by intervention were explored for efficacy.

## Methods and materials

### Protocol and registration

Conduct of this systematic review aligned with the updated Preferred Reporting Items for Systematic Reviews and Meta-Analysis (PRISMA) guidelines (14), with the protocol registered with Open Science Framework (15).

### Eligibility criteria

Inclusion criteria for the systematic review are summarized in Table 1.

**Table 1:** Inclusion criteria (PICOS).

Participants and setting	Women >18 years. They may have been diagnosed with CVD-related risk markers (abnormal blood pressure, dyslipidaemia, abnormal blood glucose) or have CVD related lifestyle risk factors (sedentary behavior, poor diet quality, overweight/obesity, or smoke).
Intervention	Primary or secondary prevention CVD interventions, targeting behavior change for ≥1 lifestyle risk factor (poor diet quality, physical inactivity/sedentary behavior, smoking, poor sleep habits, excess body weight, excessive alcohol intake). Primary prevention targeted the above lifestyle risk factors with the purpose of preventing a CVD diagnosis while secondary prevention targeted those risk factors among women diagnosed with CVD risk markers (e.g., high blood pressure), or an early diagnosis of cardiovascular morbidity.
Comparators	No-intervention, usual care or waitlist control group, or other type of lifestyle intervention.
Outcomes	CVD morbidity and mortality (CVD diagnosis, events, or mortality), risk markers (blood pressure, blood lipids, blood glucose levels) and lifestyle risk factors (dietary quality, physical activity/sedentary behavior, weight, smoking status, sleep quality, alcohol intake).
Study design	Randomized controlled trials, pseudo-randomized controlled trials and cluster randomized controlled trials.

CVD, cardiovascular disease.

### Search strategy

Six electronic databases (MEDLINE, EMBASE, PsycINFO, Web of Science, CINAHL and Cochrane Library) were searched using a pre-defined search strategy from date of inception to 4th January 2022 (Supplementary Table 1) and limited to records published in English with human subjects. Additionally, the reference lists of included studies were searched. Citations of included studies were searched in Scopus on the 10th of November 2020 and the 4th of January 2022. Lastly, field experts were contacted *via* email to ensure that all eligible studies were identified.

### Study selection

Titles, abstracts, and keywords of articles identified were screened for eligibility, and eligible full texts were retrieved and screened by two independent reviewers (K.S and M.H). Studies were excluded if they were not published in English, did not specifically target women, lifestyle risk factors and health behaviors, and were not primary or secondary prevention interventions for CVD. Disagreements in assessments between reviewers were resolved by a third reviewer (R.T). Study selection was managed using Covidence software (16).

### Data extraction

Data were extracted by one reviewer (K.S) and checked by a second reviewer (K.J.O) or (R.T) using a standardized data extraction tool developed by the authors. The following information was extracted: study characteristics (authors, date and country of publication, study setting and population), description of the intervention and comparison groups and a description of the study outcomes (outcome, measurement tool, statistically significant findings).

### Risk of bias and quality of evidence

Risk of bias for eligible studies was assessed by two independent reviewers (K.S and R.T or K.J.O) using the Cochrane Collaborations tool (17) for assessing risk of bias. Disagreements in assessments between reviewers were resolved by a third reviewer (M.H). Only RCTs were considered in the data synthesis. Each of the criteria; sequence generation, allocation concealment, blinding of participants/personnel, blinding of outcome assessment, incomplete outcome data, selective reporting and other sources of bias were rated as yes/low risk of bias, no/high risk of bias or unclear by the reviewers.

### Data synthesis

Studies were categorized by level of prevention (primary or secondary prevention) with results described overall, and by level of prevention. CVD morbidity and mortality, changes in CVD risk markers and lifestyle risk factors within included RCTs were categorized as either significant between-group changes or not statistically significant. This was based on differences reported between groups at baseline to the end of respective interventions (grouped as ≤ 6 months, 12 months, and >12 months).

For the primary aim, meta-regression was carried out via multilevel modeling to evaluate overall intervention impact by time on change in systolic blood pressure (SBP), diastolic blood pressure (DBP), total cholesterol (TC), low density lipoproteins (LDL), high density lipoproteins (HDL), triglycerides (TG), blood glucose levels (BGLs) and body mass index (BMI) using the using the rma.mv function in the metafor package version 2.1-0 (https://www.metafor-project.org) (18) in R, version 3.6.1 (R Foundation for Statistical Computing, Viennna, Austria). For each study, effects at each time point were estimated as the mean difference between groups, (Control—Treatment), as Hedges' g using the unbiased option for variance estimates. The multilevel model used two random effects fitted using restricted maximum likelihood estimation. The highest-level random effect was for study differences with an additional random effect time, nested within study for the repeated time measurements within each study arm. The significance of fixed effects in the model was assessed using the Q statistic, a Wald-type test of the model coefficients for a given model term. Total *I*^2^ for the multilevel model was calculated by the method described by Viechtbauer, (*I*^2^ for Multilevel and Multivariate Models) (19). Residual plots were used to check that the following modeling residual assumptions were met, homogeneity of variance, normality, and the absence of outliers. Unless noted otherwise the assumptions were found to be satisfactory.

The effect sizes reported were for change over time based on the difference of the mean differences between baseline and each time point, e.g., (mean difference at 6 min or less—mean difference at baseline). To assess for publication bias, funnel plots were produced and visually inspected and supported with rank correlation tests for funnel plot asymmetry. All models were checked in this way, and unless otherwise noted, satisfied this assumption (Supplementary material). For each meta-analysis, the level of evidence was applied to the GRADE (Grading of Recommendations Assessment, Development and Evaluation) criteria and reported. Effect plots with 95% confidence intervals were based on marginal mean effects from the fitted model or differences of marginal mean effects. Forest plots (Supplementary material) did not include an overall effect as there were moderator variables in the models, as individual effects might not have been from the same population.

For the secondary objective of the review, the meta-regression was extended to determine whether the time-based effects varied depending on other grouping variables. The additional fixed effect moderators tested included level of prevention (primary or secondary), and intervention treatment type, including type of lifestyle intervention i.e., diet or physical activity or diet and physical activity or diet, physical activity and smoking or diet, physical activity and weight management or diet, physical activity, sedentary behavior, and weight management, or number of interventions ranging from 1 to 4. All moderators were treated as categorical variables. Other population variables, such as history of pregnancy complications or country of study origin were not tested due to small sample sizes. These additional moderators were tested individually by adding them as main effects and as an interaction with the fixed time effect. Non-significant moderator effects were not retained. As above, residual plots were examined to test model assumptions.

## Results

### Selection of studies

**Figure 1:**
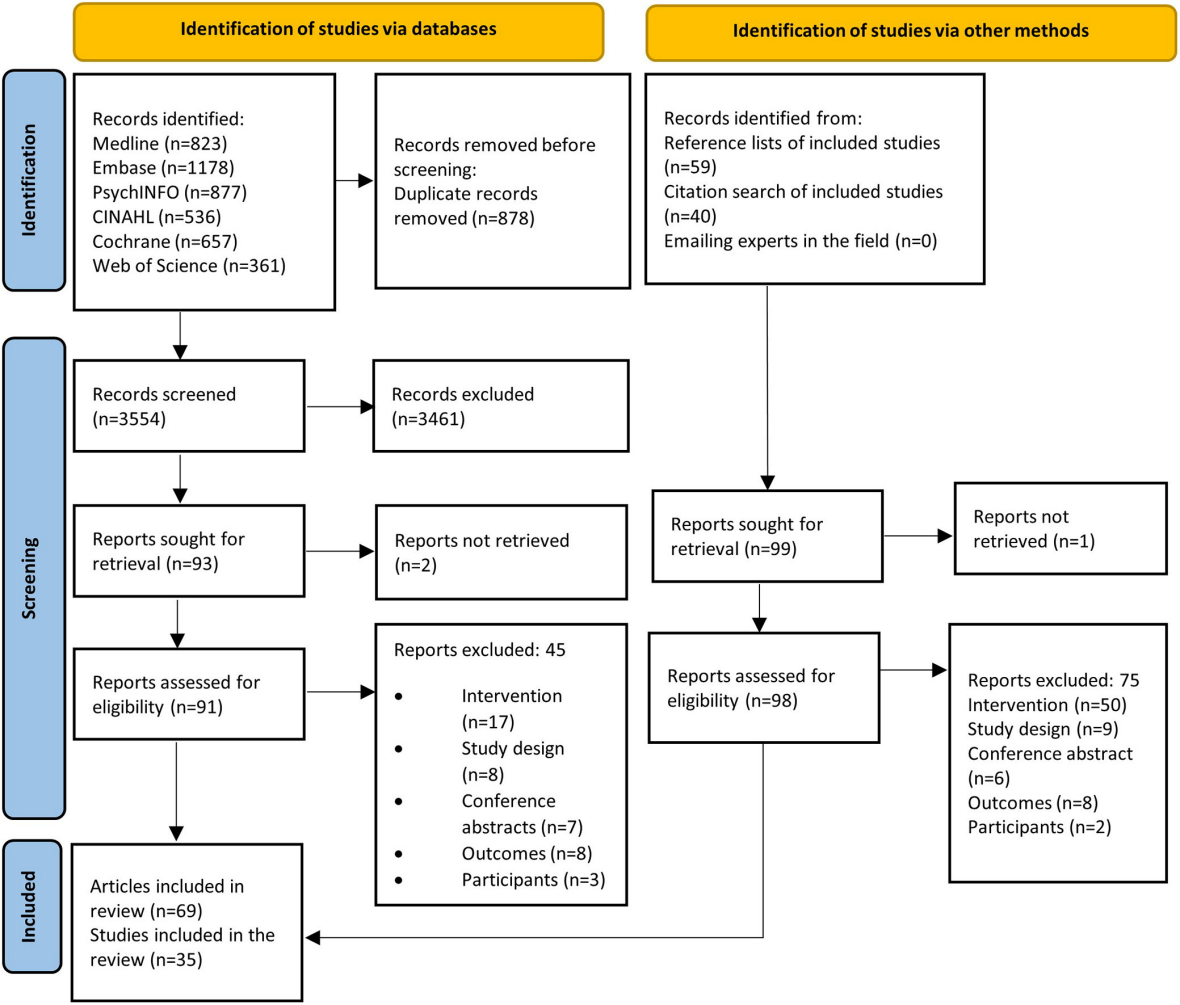
PRISMA flow diagram.

A total of 3,868 articles were identified with 35 RCTs reported across 62 articles, with 24 focused on primary CVD prevention and 11 on secondary prevention (Figure 1).

### Risk of bias assessment

**Figure 2:**
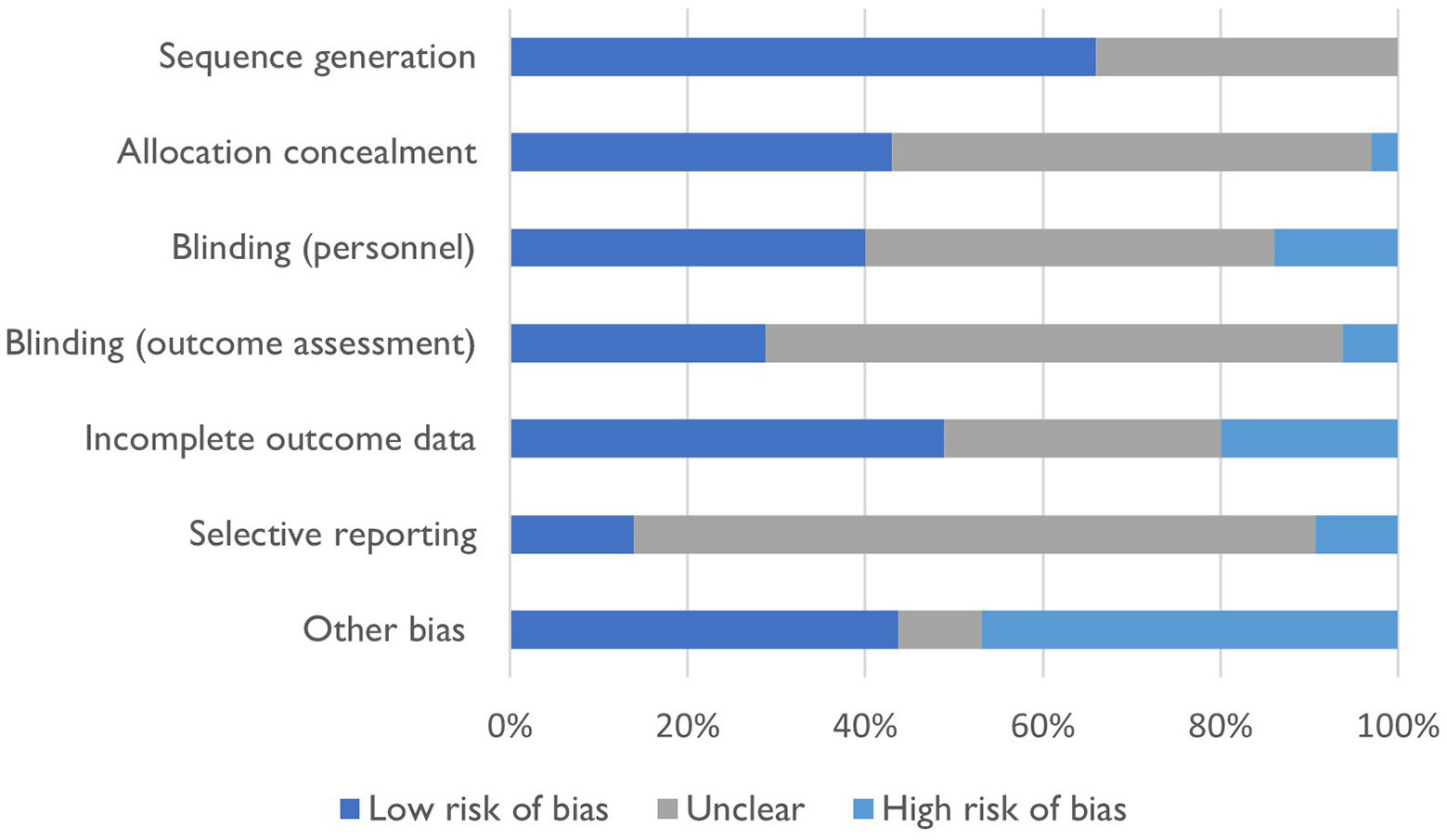
Risk of bias for included studies.

Figure 2 summarizes the risk of bias assessment for included studies. There was a low risk of bias for sequence generation and incomplete outcome assessment, with 66% of studies (*n* = 23) adequately describing sequence generation and 49% of studies (*n* = 17) adequately discussing incomplete outcome data. However, 54% of studies (*n* = 19) failed to adequately describe concealment methods for participant allocation to study groups, blinding of study personnel (*n* = 16, 46%), blinding of outcome assessment (*n* = 23, 66%) and selective outcome reporting (*n* = 27, 77%). Studies were more likely to either have a low source of other bias (*n* = 16, 46%) or a high source of other bias (*n* = 15, 43%).

### Characteristics of included studies

Study characteristics are summarized in Table 2. In summary, the majority of RCTs were carried out in the United States (USA) (*n* = 25). Primary prevention interventions were predominately from the USA (*n* = 20) with more secondary prevention interventions (*n* = 5) conducted in Asia/Middle East than primary prevention (*n* = 2). More than 50% of studies were published between 2006 and 2015. In total, 57,908 women (sample range: 14 to 48,853) were included across the 35 studies. A higher proportion of studies included middle-aged women (51–65 years) compared to women < 50 years. The majority (*n* = 31) of included studies were traditional RCTs comparing intervention groups with a “usual care” or no intervention control group.

**Table 2:** Summary of study characteristics of 35 randomized controlled trials, by level of prevention (primary or secondary).

**Characteristics**		**Primary prevention *n* (%)**	**Secondary prevention *n* (%)**	**Total *n* (%)**
**Intervention type**		24 (68.57)	11 (31.43)	35 (100)
**Number of participants**	Total (n)			57,908
	Mean			1,654.51
	Standard Deviation			8,219.87
	Median			151
	Interquartile Range			28–1,089
	Range (n)			14–48,853
**Publication year**	≤ 2000	1 (4.17)	2 (18.18)	3 (8.57)
	2001–2005	3 (12.50)	0 (0.00)	3 (8.57)
	2006–2009	8 (33.33)	1 (9.09)	9 (25.71)
	2010–2015	5 (20.83)	4 (36.36)	9 (25.71)
	≥2016	7 (29.17)	4 (36.36)	11 (31.43)
**Country of publication**	North America (US/Canada)	20 (83.33)	5 (45.45)	25 (71.43)
	South America	0 (0.00)	1 (9.09)	1 (2.86)
	Australia and New Zealand	2 (8.33)	0 (0.00)	2 (5.71)
	Asia/Middle East	2 (8.33)	5 (45.45)	7 (20.00)
**Mean age of study sample**	18–35 years	3 (12.50)	0 (0.00)	3 (8.57)
	36–50 years	7 (29.17)	1 (9.09)	8 (22.86)
	51–65 years	14 (58.33)	9 (81.82)	23 (65.71)
	>65 years	0 (0.00)	1 (9.09)	1 (2.86)
**Ethnicity/race**	≥ 80% Caucasian	11 (45.83)	2 (18.18)	13 (37.14)
	≤ 80% Caucasian	10 (41.67)	4 (36.36)	15 (42.86)
	Not reported	3 (12.50)	5 (45.45)	8 (22.86)
**Education level**	>50% with college/university degree	4 (16.67)	1 (9.09)	5 (14.29)
	< 50% with college/university degree	7 (29.17)	1 (9.09)	8 (22.86)
	Other	5 (20.83)	5 (45.45)	10 (28.57)
	Not reported	8 (333.33)	4 (36.36)	12 (34.29)
**Mode of delivery**	Online (website/emails/program)	5 (20.83)	0 (0.00)	5 (14.29)
	Face-to-face (community setting)	12 (50.00)	7 (63.64)	19 (54.29)
	Face-to-face (home-based)	2 (8.33)	0 (0.00)	2 (5.71)
	Hybrid (face-to-face and online)	5 (20.83)	4 (36.36)	9 (25.71)
Intervention deliverer*	Online program	2 (8.33)	0 (0.00)	2 (5.71)
	Trained facilitators	9 (37.50)	1 (9.09)	10 (28.57)
	Community health worker or nurse	4 (16.67)	6 (54.55)	10 (28.57)
	Exercise physiologist/physiotherapist	5 (20.83)	2 (18.18)	7 (20.00)
	Nutrition professional	9 (37.50)	2 (18.18)	11 (31.43)
	Psychologist	1 (4.17)	2 (18.18)	3 (8.57)
	Other	2 (8.33)	1 (9.09)	3 (8.57)
	Not reported	3 (12.50)	2 (18.18)	5 (14.29)
**Duration of intervention**	≤ 3 months	11 (45.83)	8 (72.73)	19 (54.29)
	>3 to ≤ 6 months	3 (12.50)	1 (9.09)	4 (11.43)
	>6 to ≤ 12 months	6 (25.00)	0 (0.00)	6 (17.14)
	>12 to ≤ 18 months	2 (8.33)	1 (9.09)	3 (8.57)
	>18 to ≤ 24 months	0 (0.00)	1 (9.09)	1 (2.86)
	≥24 months	2 (8.33)	0 (0.00)	2 (5.71)
CVD behavioral risk factors targeted*	Dietary intake	19 (79.17)	7 (63.64)	26 (82.86)
	Physical activity	22 (91.67)	8 (72.73)	30 (85.71)
	Sedentary behavior	1 (4.17)	0 (0.00)	1 (2.86)
	Sleep habits	0 (0.00)	0 (0.00)	0 (0.00)
	Alcohol intake	0 (0.00)	1 (9.09)	1 (2.86)
	Smoking habits	3 (12.50)	2 (18.18)	5 (14.29)
	Weight management	3 (12.50)	2 (18.18)	5 (14.29)
Outcomes*	Cardiovascular disease mortality	1 (4.17)	0 (0.00)	1 (2.86)
	Cardiovascular disease morbidity	1 (4.17)	1 (9.09)	2 (5.71)
	Blood Pressure	18 (75.00)	8 (72.73)	25 (71.43)
	Blood Lipids (Cholesterol, LDL, HDL, TG).^a^	17 (70.83)	7 (63.64)	24 (68.57)
	Blood Glucose Levels	15 (62.50)	4 (36.36)	19 (54.29)
	Dietary Intake	15 (62.50)	3 (27.27)	18 (51.43)
	Physical Activity	21 (87.50)	5 (45.45)	25 (71.43)
	Sedentary Behavior	3 (12.50)	0 (0.00)	3 (8.57)
	Smoking behavior	2 (8.33)	4 (36.36)	6 (17.14)
	Sleep quality	0 (0.00)	0 (0.00)	0 (0.00)
	BMI^a^	22 (91.67)	8 (72.73)	30 (85.71)

*Categories are not mutually exclusive.

^a^LDL (low density lipoproteins), HDL (high density lipoproteins), TG (triglycerides), BMI (body mass index).

### Interventions

Of the 24 primary prevention interventions, dietary intake (*n* = 19) and physical activity (*n* = 22) were the most common lifestyle risk factors targeted, which was similar for the 11 secondary prevention interventions (*n* = 7 and *n* = 8, respectively). The majority of studies targeted both dietary intake and physical activity [17 primary (20–36) and six secondary interventions (37–42)]. Interventions were predominately delivered in a face-to-face format in group settings for primary prevention (*n* = 12) and secondary prevention (*n* = 7) interventions. Majority of the interventions lasted ≤ 12 months (primary prevention *n* = 20 and secondary prevention *n* = 9), and of those, 19 studies lasted ≤ 3 months (primary prevention *n* = 11 and secondary prevention *n* = 8).

### Outcomes

The outcomes from both primary and secondary prevention interventions are presented in Table 3.

**Table 3:** Summary of interventions and outcomes reported by studies (*n* = 35) included in the review.

	**Lifestyle risk factors^§^**	**CVD mortality**	**CVD morbidity**	**Blood pressure**	**Blood lipids**	**Blood glucose**	**Dietary intake**	**Physical activity**	**Sedentary behavior**	**Smoking status**	**Sleep quality**	**Body mass index**
Beckie et al. (43)^b^	PA	Ø	Ø	Ø	Ø	Ø	Ø	**+**	Ø	Ø	Ø	Ø
Chee et al. (44)^a^	PA	Ø	Ø	Ø	Ø	Ø	Ø	**+**	Ø	Ø	Ø	Ø
Cornélio et al. (45)^b^	D	Ø	Ø	Ø	Ø	Ø	+^*^	Ø	Ø	Ø	Ø	Ø
Folta, (26)^a^	D, PA	Ø	Ø	Ø	Ø	Ø	+^*^	+^*^	Ø	Ø	Ø	+^*^
Hageman, (29)^a^	D, PA	Ø	Ø	+^*^	**+**	**+**	+^*^	**+**	Ø	Ø	Ø	**+**
Hayashi, (37)^b^	D, PA	Ø	Ø	+^*^	**+**	Ø	+^*^	+^*^	Ø	**+**	Ø	**+**
Howard, (46)^a^	D	**+**	**+**	+^*^	+^*^	Ø	Ø	Ø	Ø	Ø	Ø	+^*^
Hutchesson, (20)^a^	D, PA, SB, W	Ø	Ø	**+**	**+**	**+**	**+**	**+**	**+**	Ø	Ø	**+**
Hwang et al. (47)^a^	D	Ø	Ø	**+**	**+**	**+**	Ø	Ø	Ø	Ø	Ø	**+**
Keyserling, (23)^a^	D, PA	Ø	Ø	**+**	**+**	**+**	+^*^	+^*^	Ø	Ø	Ø	**+**
Khare, (21)^a^	D, PA	Ø	Ø	**+**	**+**	**+**	+^*^	+^*^	Ø	Ø	Ø	**+**
Khare, (22)^a^	D, PA	Ø	Ø	**+**	**+**	**+**	+^*^	+^*^	Ø	Ø	Ø	+^*^
Kuller, (30)^a^	D, PA, W	Ø	Ø	**+**	+^*^	+^*^	+^*^	+^*^	Ø	Ø	Ø	+^*^
Lawton, (48)^a^	PA	Ø	Ø	**+**	**+**	**+**	Ø	+^*^	Ø	Ø	Ø	**+**
Lin, (41)^b^	D, PA	Ø	Ø	**+**	+^*^	**+**	Ø	+^*^	Ø	Ø	Ø	Ø
Low, (33)^a^	D, PA, S	Ø	Ø	Ø	Ø	Ø	Ø	**+**	Ø	Ø	Ø	**+**
Mosca, (42)^b^	D, PA, S, W	Ø	Ø	**+**	**+**	Ø	Ø	**+**	Ø	**+**	Ø	**+**
Oh, (39)^b^	D, PA	Ø	Ø	**+**	+^*^	**+**	Ø	Ø	Ø	Ø	Ø	+^*^
Oh, (40)^b^	D, PA	Ø	Ø	**+**	**+**	**+**	Ø	Ø	Ø	Ø	Ø	+^*^
Pazoki et al. (49)^a^	PA, A	Ø	Ø	+^*^	**+**	**+**	Ø	+^*^	Ø	Ø	Ø	**+**
Perry et al. (50)^a^	PA	Ø	Ø	Ø	Ø	Ø	Ø	**+**	Ø	Ø	Ø	Ø
Rich-Edwards, (34)^a^	D, PA, S	Ø	Ø	**+**	Ø	Ø	**+**	**+**	+^*^	Ø	Ø	**+**
Schmitz, (51)^b^	S	Ø	Ø	Ø	Ø	Ø	Ø	Ø	Ø	**+**	Ø	Ø
Seguin, (27)^a^	D, PA	Ø	Ø	**+**	+^*^	**+**	**+**	+^*^	Ø	Ø	Ø	+^*^
Sequin-Fowler, (28)^a^	D, PA	Ø	Ø	**+**	**+**	**+**	+^*^	+^*^	**+**	Ø	Ø	+^*^
Simkin-Silverman, (32)^a^	D, PA, W	Ø	Ø	+^*^	+^*^	+^*^	+^*^	+^*^	Ø	+^*^	Ø	+^*^
Staffileno, (52)^a^	PA	Ø	Ø	+^*^	Ø	Ø	Ø	**+**	Ø	Ø	Ø	Ø
Staffileno, (35)^a^	D, PA	Ø	Ø	**+**	Ø	Ø	+^*^	**+**	Ø	Ø	Ø	**+**
Stoddard, (24)^a^	D, PA	Ø	Ø	**+**	**+**	Ø	**+**	+^*^	Ø	Ø	Ø	Ø
Taha et al. (53)^b^	PA	Ø	Ø	**+**	Ø	Ø	Ø	Ø	Ø	Ø	Ø	**+**
Toobert, (38)^b^	D, PA	Ø	+^*^	**+**	**+**	Ø	+^*^	+^*^	Ø	**+**	Ø	+^*^
Toobert, (31)^a^	D, PA, S	Ø	Ø	Ø	Ø	Ø	+^*^	+^*^	Ø	Ø	Ø	+^*^
Tsai, (54)^b^	D, PA	Ø	Ø	**+**	+^*^	**+**	Ø	Ø	Ø	Ø	Ø	**+**
Witmer, (25)^a^	D, PA, S	Ø	Ø	**+**	**+**	Ø	+^*^	+^*^	Ø	**+**	Ø	**+**
Wu, (36)^a^	D, PA	Ø	Ø	+^*^	+^*^	**+**	Ø	Ø	Ø	Ø	Ø	+^*^

**+**, Outcome within the study; Ø, Not an outcome within the study.

^a^Primary prevention intervention, ^b^Secondary prevention intervention.

*Results from the outcome were statistically significant between the intervention arm and the control arm (p < 0.05).

^§^Lifestyle risk factors targeted within the study: D (diet), PA (physical activity), SB (sedentary behavior), S (smoking), A (alcohol intake), SP (sleep quality), W (weight management).

#### Cardiovascular related morbidity and mortality

One study reported both CVD mortality and morbidity (46). The 24-month (*n* = 48,853) primary prevention study compared face-to-face group sessions on dietary improvements with a no-intervention control and found no significant difference in numbers of fatal and non-fatal CVD (including stroke).

#### Cardiovascular risk markers

Cardiovascular risk markers (SBP, DBP, TC, LDL-C, HDL-C, TG and BGLs) and BMI were combined in a meta-analysis. The types of outcomes included in the meta-analysis, the statistical significance reported by each study and total heterogeneity are displayed in Table 4. Across SBP, DBP, TC, LDL, HDL, BGLs and BMI, the *I*^2^ ranged from 76 to 99%, signifying large heterogeneity between studies. Studies reporting TG showed a smaller amount of heterogeneity between studies (*I*^2^=45%). Plots of the mean difference of study effects over time, forest plots for the individual study effects, residual diagnostic plots, and funnel plots to assess bias are provided in Supplementary materials for each outcome.

**Table 4:** Meta-analysis effect sizes (Control-Treatment) for change from baseline at 3 time points, statistical significance, and total heterogeneity.

**Measure**	**Time**	**Effect**	**95% CI**	**Q (3)**	***p***	**Total *I*^2^ (%)**
SBP	6 min or less	3.52	(1.57, 5.46)	13.0	0.005	99
	12 min	0.90	(−1.36, 3.15)			
	>12 min	0.95	(−1.83, 3.73)			
DBP	6 min or less	−0.25	(−1.35, 0.84)	1.52	0.68	99
	12 min	−0.33	(−1.55, 0.89)			
	>12 min	−0.92	(−2.41, 0.57)			
TC	6 min or less	3.11	(−1.68, 7.90)	2.16	0.54	98
	12 min	−0.72	(−5.65, 4.21)			
	>12 min	0.71	(−4.81, 6.24)			
LDL_C	6 min or less	0.99	(−3.58, 5.56)	0.85	0.84	95
	12 min	−1.34	(−6.88, 4.21)			
	>12 min	1.47	(−4.61, 7.56)			
HDL_C	6 min or less	−0.31	(−1.68, 1.06)	1.61	0.66	95
	12 min	0.29	(−1.17, 1.74)			
	>12 min	0.78	(−0.88, 2.44)			
TG	6 min or less	5.69	(−0.56, 11.93)	3.77	0.29	45
	12 min	2.37	(−7.06, 11.79)			
	>12 min	−0.88	(−7.41, 5.66)			
BGLs	6 min or less	3.15	(0.16, 6.14)	4.88	0.18	96
	12 min	2.33	(−1.37, 6.02)			
	>12 min	0.52	(−3.25, 4.29)			
BMI	6 min or less	0.95	(0.54, 1.35)	21.6	< 0.001	76
	12 min	0.61	(0.07, 1.16)			
	>12 min	0.58	(0.01, 1.16)			

SBP: systolic blood pressure, DBP: diastolic blood pressure, TC: total cholesterol, LDL-C: low-density lipoprotein cholesterol, HDL-C: high-density lipoprotein cholesterol, TG: triglycerides, BGL: blood glucose levels, BMI: body mass index. 95% CI: 95% confidence interval.

For all measures, a positive value indicates improvement, except for measure HDL-C where a negative value indicates improvement.

#### Cardiovascular risk markers: Blood pressure

Of the 25 studies that assessed blood pressure (BP), there were statistically significant decreases in BP in the intervention group compared with the control group for seven primary prevention studies (29, 30, 32, 36, 46, 49, 52) and one secondary prevention study (37). Of these, five targeted diet and physical activity (29, 30, 32, 36, 37), and two also targeted weight management (30, 32). Of the other three studies, two targeted physical activity (49, 52) and the other diet only (46). In addition, seven of these studies followed a face-to-face intervention (group or individual) (29, 30, 32, 37, 46, 49, 52) and six studies lasted ≤ 12 months duration (29, 30, 32, 36, 49, 52).

Twenty of these studies were combined in a meta-analysis. Compared to control groups, the intervention groups achieved significantly greater reductions in mean SBP (mmHg) at ≤ 6 months (3.52, *p* = 0.005), based on data from 2,380 participants in 19 RCTs. However, the outcome was downgraded from high to moderate quality because there was serious inconsistency across studies (Supplementary Table 3). Results did not achieve statistical significance for DBP (mmHg) at (2.25, *p* = 0.65) ≤ 6 months, both SBP or DBP at 12 months (0.90, *p* = 0.44 and −0.33, *p* = 0.60) or SBP >12 months (0.95, *p* = 0.50), and these outcomes were all downgraded from high to low quality evidence because there was serious inconsistency and imprecision across studies. Results also did not achieve statistical significance for DBP >12 months (−0.92, *p* = 0.23), and the outcome was downgraded to moderate quality due to serious inconsistency across studies.

**Figure 3:**
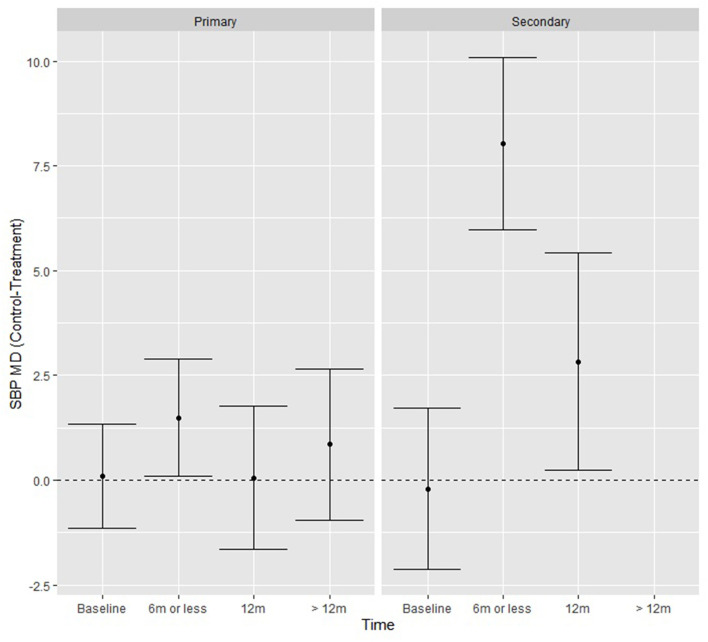
SBP fitted model means from the moderation analysis showing the effect of the time by study type interaction, where secondary prevention studies had significantly greater reductions in SBP at ≤ 6 and 12 months.

When exploring additional moderators, (level of prevention, control and treatment type, number of lifestyle interventions and lifestyle interventions present), there was a significant study type by time interaction Q (2) = 9.28, p = 0.001 in SBP only. Compared with the control groups, the intervention groups achieved significantly greater reductions in mean SBP (mm Hg) at ≤ 6 months (8.03, *p* < 0.01) and at 12 months (2.83, *p* = 0.03) (Figure 3), if they were secondary CVD prevention studies.

#### Cardiovascular risk markers: Blood lipids

Of the 24 studies that measured blood lipids, there were statistically significant improvements in one or more measures of lipid profiles (TC, LDL, HDL, TG) in the intervention group compared with the control group for eight studies (five primary prevention, three secondary prevention) (27, 30, 32, 36, 39, 41, 46, 54). Four of the primary prevention (27, 30, 32, 36) and two secondary prevention interventions (41, 54) targeted both diet and physical activity in their interventions. Seven of the eight studies followed a face-to-face (group or individual) intervention (27, 30, 32, 39, 41, 46, 54) and seven studies lasted ≤ 12 months in duration (27, 32, 36, 39, 41, 54). Of these eight studies, four also observed statistically significant decreases in BP between groups (30, 32, 36, 46).

Results from the meta-analyses at of TC (*n* = 16), LDL-C (*n* = 14), HDL-C (*n* = 15) and TG (*n* = 12) demonstrated no significant difference between groups at ≤ 6 months, 12 months and >12 months in mean (mg/dL) TC (3.11, *p* = 0.20, −0.72, *p* = 0.77 and 0.71, *p* = 0.80, respectively), LDL-C (0.99, *p* = 0.67, −1.34, *p* = 0.64 and 1.47, *p* = 0.63, respectively), HDL-C (−0.31, *p* = 0.66, 0.29, *p* = 0.70 and 0.78, *p* = 0.36, respectively) and TG (5.69, *p* = 0.07, 2.37, *p* = 0.62 and −0.88, *p* = 0.79, respectively). TC, LDL-C, HDL-C and TG were downgraded from high to moderate quality evidence at >12 months due to serious inconsistency across studies (Supplementary Tables 4, 5). TC was downgraded from high to low quality evidence at ≤ 6 months and 12 months due to serious inconsistency and imprecision across studies. LDL-C, HDL-C and TG were downgraded from high to very low-quality evidence at ≤ 6 months and 12 months due to serious inconsistency and very serious imprecision across studies.

When exploring additional moderators (level of prevention, control and treatment type, number of lifestyle interventions and lifestyle interventions present), results did not reach significance for all additional moderators. When exploring residual plots for both TC and TG, there was presence of extreme outliers. The funnel plot for TC was generally symmetrical, and although Kendall's Tau rank correlation test (τ = −0.24, *p* = 0.02) is weakly significant. there is no strong indication of publication bias.

#### Cardiovascular risk markers: Blood glucose levels

Of the 19 studies that measured BGLs, results were statistically significant for two primary prevention studies (30, 32). Both targeted diet, physical activity, and weight management (30, 32). One study lasted ≤ 12 months (32). Both studies were conducted face-to-face in a group setting.

Fifteen of these studies were combined in a meta-analysis, which demonstrated significantly greater reductions in mean BGLs (mg/dL) at ≤ 6 months (3.15, *p* = 0.04) between intervention and control groups, but not at 12 months (2.33, *p* = 0.22) or > 12 months (0.52, *p* = 0.79), however the overall Q test was not statistically significant (Table 4). The outcome was downgraded from high to moderate quality evidence at ≤ 6 months and >12 months due to serious inconsistency across studies, whereas at 12 months it was downgraded to low quality evidence due to serious inconsistency and imprecision across studies (Supplementary Table 6).

**Figure 4:**
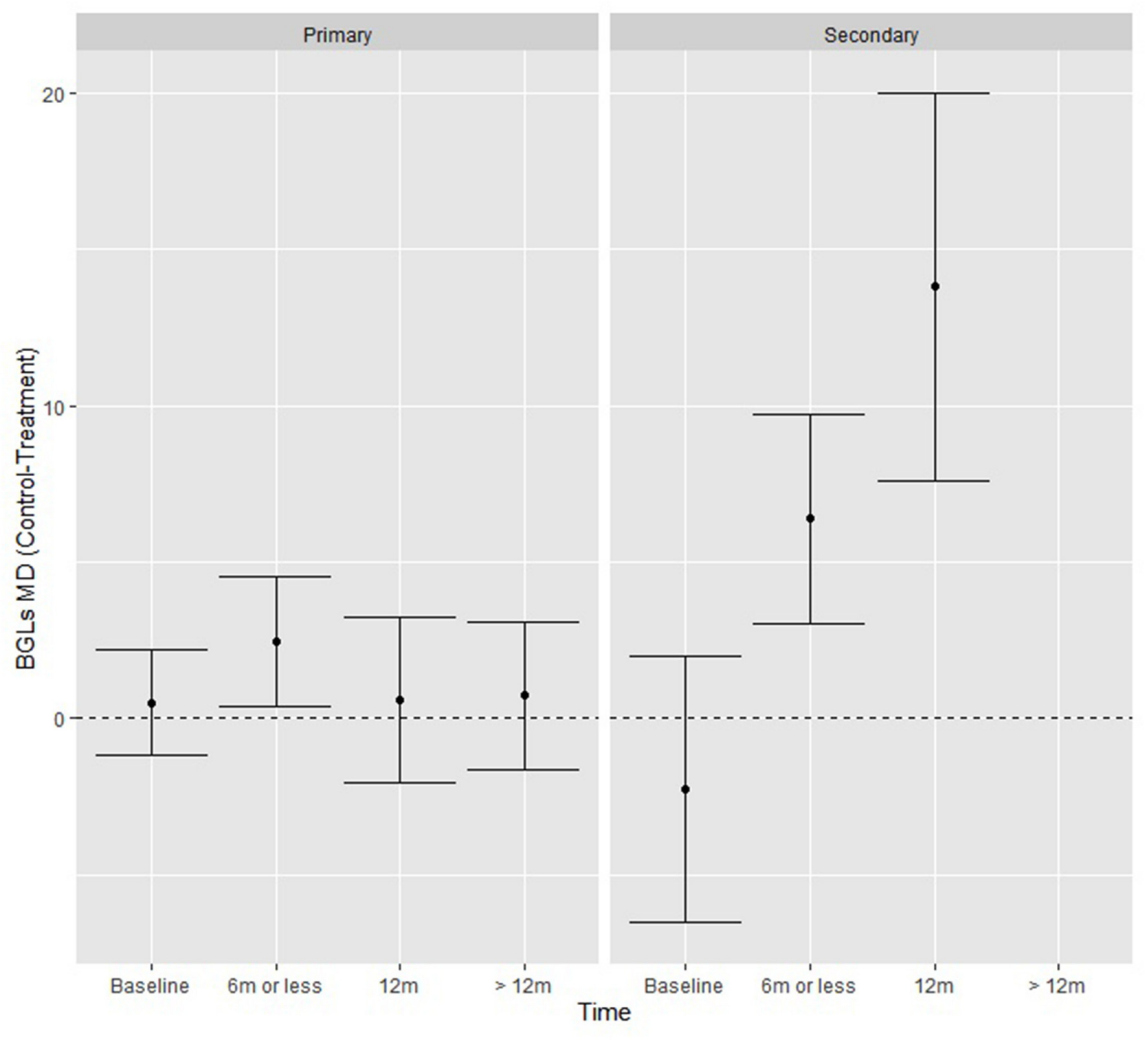
BGL fitted model means from the moderation analysis showing the effect of the time by study type interaction, where secondary prevention studies had significantly greater reductions in BGLs at each time-point.

When testing additional moderators (level of prevention, control and treatment type, number of lifestyle interventions and lifestyle interventions present), there was a significant study type by time interaction Q (2) = 15.13, *p* < 0.001, where there was no difference over time for primary study types, but for secondary types the intervention groups achieved significantly greater reductions relative to control in mean BGLs (mg/dL) at ≤ 6 months (6.38, *p* < 0.01) and at 12 months (13.80, *p* < 0.01) (Figure 4). The funnel plot was generally symmetrical, and although Kendall's Tau rank correlation test (τ = −0.22, *p* = 0.04) is weakly significant there is no strong indication of publication bias.

#### Body mass index

BMI was an outcome for 30 of the studies, where 23 of the 30 studies used a combination of diet and physical activity as interventions within their studies and 12 studies saw statistically significant improvements in BMI in the intervention group compared to control (22, 26–28, 30–32, 36, 38–40, 46). Of these 12 studies, all but one (36) were delivered in a face-to-face group setting and 10 lasted ≤ 12 months in duration (22, 26–28, 30, 32, 36, 38–40).

Of these 30 studies, 18 studies that reported BMI at each time point for intervention and comparison groups were combined in a meta-analysis. Results from this meta-analysis showed that there was a significant change over time, where the intervention groups achieved significantly greater reductions in mean BMI (kg/m2) relative to the control group at ≤ 6 months (0.95, *p* < 0.01) based on data from 2232 participants in 18 RCTs, at 12 months (0.61, *p* = 0.03) from 1,865 participants in 8 RCTs and at ≥ 12 months (0.58, *p* = 0.05) from 45096 participants in 4 RCTs. At ≤ 6 months the outcome was downgraded from high to moderate quality evidence due to serious inconsistency across studies, whereas at 12 months it was downgraded to low quality evidence due to serious inconsistency and imprecision across studies. At >12 months, the outcome remained high quality evidence (Supplementary Table 6).

When exploring additional moderators (level of prevention, control and treatment type, number of lifestyle interventions and lifestyle interventions present) results did not reach significance.

#### Lifestyle factors

Of the 18 studies that reported dietary intake as an outcome, improvements in measures of dietary intake were statistically significant in the intervention group compared to the controls for 13 studies (10 primary and three secondary prevention interventions) (21–23, 26, 28–32, 35, 37, 38, 45). Improvements in measures of physical activity were targeted in 25 studies, with 16 reporting statistically significant increases in physical activity in the intervention group compared to control (13 primary prevention and three secondary) (21–28, 30–32, 37, 38, 41, 48, 49). Of these, two used only physical activity as an intervention (48, 49), whereas the other 14 used a combination of diet and physical activity to prevent CVD. Ten studies reported statistically significant improvements in various measures of both dietary intake and physical activity (eight primary and two secondary prevention interventions) (21–23, 26, 28, 30–32, 37, 38). Of these studies, all were delivered in a face-to-face group setting, all included participants ≥40 years old and seven had interventions lasting ≤ 12 months in duration (21–23, 26, 28, 31, 32). Of the six studies that reported a reduction in smoking as an outcome (two primary and four secondary prevention interventions) (25, 32, 37, 38, 42, 51), one study saw the intervention group report a statistically significant decrease in the daily number of cigarettes smoked compared to the control group (32). A reduction in sedentary behavior was reported by three studies, all with primary prevention interventions (20, 28, 34), where one saw a statistically significant decrease in time spent inactive (watching television, reading or on the computer) in the intervention group compared with the control (34). There were no studies that reported improvements in sleep quality as an outcome. Lifestyle risk factors were not included in the meta-analysis due to inconsistency with outcome measures.

## Discussion

The current systematic review includes 35 RCTs and is the first to synthesize evidence on the effectiveness of lifestyle interventions specifically targeting women for primary and secondary CVD prevention. There was high-level evidence for interventions targeting BMI reduction at >12 months and a moderate level of evidence for lowering SBP, BGLs and BMI at ≤ 6 months, BGLs at 12 months and DBP, TC, LDL-C, HDL-C, TG and BGLs at >12 months. Meta-analyses conducted in the current review demonstrated that lifestyle CVD prevention interventions achieved statistically significant reductions in BMI, BGLs and SBP compared with usual care. Sensitivity analyses, where additional moderators were tested, revealed that compared to the control groups, intervention groups in secondary prevention studies achieved significantly greater reductions in SBP and BGLs at ≤ 6 and 12 months, but not in primary prevention interventions. Other moderators (control and treatment type, number of lifestyle interventions and lifestyle interventions present) did not identify statistically significant differences between groups. Additionally, there was considerable total heterogeneity amongst outcomes included in the meta-analysis, with the exception of TG, which showed moderate heterogeneity.

The review findings indicate that lifestyle interventions can reduce CVD risk markers, specifically SBP at ≤ 6 months. However, at 12 and >12 months results were no longer significant. Interestingly, results for DBP did not reach significance before or after the sensitivity analysis at any time point. Flint et al. suggested that while SBP elevation had a greater effect on outcomes in a multivariate Cox survival analysis of data from 1.3 million adults, both SBP and DBP independently influenced CVD risk (55). However, results from this study suggests that lifestyle interventions are effective for reducing SBP in the short-term. This review could not conclude whether lifestyle interventions also reduce SBP in the long-term, due follow-up periods shorter than 12 months. Interestingly though, a systematic review and meta-analysis of lifestyle interventions (*n* = 79) for CVD prevention among female and male adults found that improvements in SBP between intervention and control groups remained statistically significant in follow-up period of ≥24 months (56). Therefore, there is a need for more research with longer-term follow-up to confirm the sustainability of SBP reduction overtime and interventions that effectively target DBP reduction for CVD prevention in women.

The meta-analysis also identified a modest but significant decrease in BGLs at ≤ 6 months, with no significant differences seen at the 12 and >12-month time-points. However, whilst the *Q*-test for BGLs displayed no significant differences between control and treatment, the confidence intervals at < 6 months were significant. This suggests that lifestyle interventions may be important to reduce BGLs at ≤ 6 months, however this advice should be adhered to with caution. The sensitivity analysis also revealed that compared to primary, the secondary prevention interventions resulted in statistically significant improvements in BGLs. These findings for both SBP and BGLs may be indicative of the initial momentum that participants present when beginning a lifestyle intervention, and the support received by study personnel throughout the intervention. For sustainable and continued progress after an initial intervention has ceased, participants not only need to maintain personal motivation, but also require environmental and social support to maintain behaviors (57). Maintenance interventions are required to determine how behaviors learned within these trials can be adopted in every-day lives (58, 59). Results from the meta-analysis of plasma lipids (TC, HDL-C, LDL-C and TG) did not reach statistical significance at any time-points, or within the sensitivity analysis. Among the RCTs that focused on dietary improvements, none specifically targeted improvements in blood lipids (e.g., to lower plasma cholesterol), and long-term follow-up of plasma lipids was only seen amongst four RCTs of very low-quality evidence. Additionally, where improvements in blood pressure and BGLs can be observed in the short-term, the success of lipid lowering, utilizing lifestyle interventions without pharmacological intervention varies widely and therefore interventions with longer follow-up periods (>12 months) may be required to detect improvements in blood lipids using lifestyle risk factors.

BMI was the only outcome included in the meta-analysis that reached statistical significance in the intervention group compared with the control at every follow-up time point (≤ 6, 12 and >12 months). The largest improvement (0.95 kg/m^2^) was seen at ≤ 6 months across evidence from 18 RCTs of moderate quality. Whereas at >12 months there was a smaller but still significant difference (0.58 kg/m^2^) from high quality evidence between groups, suggesting that statistically significant improvements in BMI can be maintained overtime. Notably, only four RCTs included weight management as a specific intervention strategy and of these, only two were included in the meta-analysis. Consequently, it appears that lifestyle interventions irrespective of providing specific advice on weight management can result in positive improvements in BMI (60). A recent meta-analysis of 31 RCTs investigating the intensity and duration of lifestyle interventions for long-term weight loss also identified that at 12 months, body weight was lower in the intervention group compared with the control (3.63 kg, 95% CI 2.58–4.67), which remained significant at 3 years (2.45 kg, 95% CI 1.17–3.73) (60). Therefore, results from the meta-analysis of low, moderate, and high-quality evidence suggest that lifestyle interventions are successful at lowering BMI in women, an important risk factor for CVD. Future studies would benefit from including weight management as a targeted intervention for CVD prevention and focusing on long-term follow-up (>12 months) to observe maintenance of results over time.

Interestingly, just one study reported on CVD morbidity and mortality, and therefore despite the review aiming to understand primary and secondary CVD prevention in women, it is unclear whether these interventions can prevent progression to CVD. Additionally, there are very few studies that use consistent measurements for outcomes of lifestyle risk factors, for example a food frequency questionnaire vs. a diet history to assess improvements in diet quality. Therefore, it was not possible to include lifestyle risk factors in quantitative data analysis. In addition, there were no studies that included interventions targeting sleep habits, which is surprising given the evidence of association between poor sleep quality and CVD and the indication of poorer sleep quality in females compared to males (61, 62). Additionally, only 11 of the 35 studies included in this review engaged participants aged ≤ 50 years old, meaning authors were unable to perform a meta-analysis using age as a moderator. It is understood that women have female-specific CVD risk factors, many of which occur and are detected in their child-bearing years. Despite this, CVD research still appears to be targeted to older women. This may be due to older literature (most studies predated 2016), and the somewhat recent understanding of the relationship between female-specific risk factors and CVD, or alternatively due to the need for long-term cohort studies to follow-up on CVD progression. Nevertheless, given that recent data suggests the stagnation of improvements in CVD risk in women < 55 years old, it is imperative that CVD interventions are also tailored to childbearing women with or without female-specific risk factors (63). A recent review of women's health studies published between 2010 and 2020 demonstrated that only 5% of studies specifically focus on CVD prevention in women, suggesting that research is disregarding the major burden of disease CVD plays in women (64). Lastly, a majority of the interventions took place in community face-to-face settings, and none within respective health services or primary care e.g., within general practice. This is surprising given a General Practitioner's (GPs) role in the prevention and management of CVD, and CVD preventative guidelines which target GPs (65, 66).

The risk of bias and GRADE assessments identified strengths and limitations within included studies. While all studies included in this review were RCTs, few studies provided detailed explanations of allocation concealment and blinding of participants and personnel. Although, given the nature of the interventions, it would not have been practical to blind participants as they were required to participate in respective activities. When assessing the quality of evidence using the GRADE approach, it was evident that most outcomes were moderate or low quality, primarily due to inconsistency of results, wide variance of point estimates across studies and minimal overlap of confidence intervals. Additionally, according to GRADE, studies within this review would normally have a serious risk of indirectness with varying populations and interventions. However, given the aims of the current review were to assess different populations and interventions, outcomes were not downgraded due to indirectness as this would not affect the quality of this review. Moreover, most of the studies utilized face-to-face group sessions and were dated ≤ 2015, suggesting that these interventions, despite reporting important findings, raise concern from an implementation perspective, given the increasing projection to e-health.

The novelty of the current review, being the first systematic review of RCTs of primary and secondary CVD interventions targeting lifestyle risk factors in women is an important strength. Other strengths of this review were the use of a comprehensive search strategy, two independent reviewers at each step of the review, and robust statistical analysis including a meta-analysis and the GRADE approach for assessing the quality of studies in the meta-analysis (67). Finally, although this systematic review and meta-analysis provides a comprehensive evaluation of a range of lifestyle interventions specifically targeted to CVD prevention, it only addresses the overall efficacy of lifestyle interventions and not individual components. From the current pool of evidence, it was not possible to determine components of the interventions that led to success (e.g., setting, delivery mode, behavior change techniques). Therefore, future research needs to address how to effectively implement CVD prevention for women. Lifestyle risk factors were unable to be included as outcomes within the meta-analysis due to the inconsistency with how they were reported and measured. Authors also acknowledge that the search strategy used in this review was designed to identify all RCTs that evaluated lifestyle interventions, also classified as CVD prevention interventions for women, irrespective of age or health status. Therefore, the search may have missed RCTs that evaluated lifestyle interventions for specific groups of women e.g., with sex-specific risk factors, which did not have the sole purpose of preventing CVD. Despite this, the search still identified two studies focused on CVD prevention after HDP, although there were no studies that reported on gravidity or parity. A future review focusing entirely on lifestyle interventions in women with sex-specific risk factors e.g., pregnancy complications, would be an important addition to the current literature. Nevertheless, this review is still an important step toward understanding which CVD risk markers can be improved with lifestyle interventions in general for the primary and secondary prevention of CVD in women.

### Recommendations for future research and practice

Lifestyle interventions aimed at preventing primary and secondary CVD in women show modest but clinically important reductions in CVD risk markers. While the current review did not reveal a superior intervention type for the prevention of CVD in women, the findings do indicate that improving lifestyle risk factors can in turn reduce a woman's risk of CVD risk markers, specifically SBP and BMI in < 12 months, and maintain improvements in BMI beyond 12 months, albeit from mostly low and moderate quality evidence. As lifestyle interventions significantly reduced BMI in women, including an aspect of weight management as a component of these lifestyle interventions may further assist with the prevention of CVD. The current systematic review and meta-analysis provide a basis for future clinical trials to continue assessing the efficacy of lifestyle risk factors for CVD prevention in women. However, there is a need to recruit younger women of childbearing age, utilize respective health services and primary care settings and provide longer follow-up to observe progression to CVD after lifestyle interventions.

## Data availability statement

The original contributions presented in the study are included in the article/Supplementary material, further inquiries can be directed to the corresponding author.

## Author contributions

KS, RT, CC, and MH were involved in the conceptualization and writing of this manuscript, as well as access to data presented in this manuscript. KC assisted with the data-analysis, meta-analysis, and writing on the data-analysis section of this manuscript. All authors contributed to the article and approved the submitted version.

## Funding

CC was supported by a National Health and Medical Research Council of Australia Leadership in Research Fellowship (APP2009340).

## Conflict of interest

The authors declare that the research was conducted in the absence of any commercial or financial relationships that could be construed as a potential conflict of interest.

## Publisher's note

All claims expressed in this article are solely those of the authors and do not necessarily represent those of their affiliated organizations, or those of the publisher, the editors and the reviewers. Any product that may be evaluated in this article, or claim that may be made by its manufacturer, is not guaranteed or endorsed by the publisher.
