# Supplementary material for: Primary and secondary cardiovascular disease prevention interventions targeting lifestyle risk factors in women: A systematic review and meta-analysis
Source: Front Cardiovasc Med. 2022 Nov 9;9:1010528. doi: 10.3389/fcvm.2022.1010528 (PMC9681924; doi:10.3389/fcvm.2022.1010528)
Supplement: Supplementary file 1 [file Data_Sheet_1.docx]

**Supplementary Material**

**Supplementary Table 1: Example Peer-Review Article Search and Update**

| **ac#** | **Searches** | **Results** |
| --- | --- | --- |
| 1 | women.mp. or Women/ | 995184 |
| 2 | female*.mp. or Female/ | 8943091 |
| 3 | 1 or 2 | 9081124 |
| 4 | male/ | 8609835 |
| 5 | 3 not 4 | 3225221 |
| 6 | cardiovascular diseases/ or cardiovascular abnormalities/ or heart defects, congenital/ or exp heart diseases/ or pregnancy complications, cardiovascular/ or exp vascular diseases/ | 2387097 |
| 7 | Stroke/ | 102501 |
| 8 | Acute Coronary Syndrome/ or (blood pressure or hypertension or blood lipids or cholesterol or triglycerides or blood glucose or blood sugar or insulin or acute coronary syndrome*).mp. | 1528285 |
| 9 | 6 or 7 or 8 | 3390947 |
| 10 | clinical trials as topic/ or randomized controlled trials as topic/ | 324834 |
| 11 | random*.tw. | 1157174 |
| 12 | group*.tw. | 3756178 |
| 13 | trial*.tw. | 1049129 |
| 14 | 10 or 11 or 12 or 13 | 4971431 |
| 15 | Risk Factors/ or risk factor*.mp. | 1142184 |
| 16 | Health Behavior/ or health behavio*.mp. | 65498 |
| 17 | Diet/ or diet*.mp. | 759687 |
| 18 | Nutrition Therapy/ or nutrition*.mp. | 390447 |
| 19 | Food/ or food*.mp. | 634014 |
| 20 | Body Weight/ or Feeding Behavior/ or Eating/ or eating behavio*.mp. | 303998 |
| 21 | Exercise Therapy/ or Leisure Activities/ or physical activit*.mp. or Exercise/ | 225498 |
| 22 | Sedentary Behavior/ or sedentary behavio*.mp. | 12990 |
| 23 | Motor Activity/ or sitting time.mp. | 97834 |
| 24 | Smoking Cessation/ or Smoking/ or "Tobacco Use Disorder"/ or smok*.mp. | 333533 |
| 25 | Nicotine/ or nicotine*.mp. | 48157 |
| 26 | "Tobacco Use"/ or "Tobacco Use Disorder"/ or Tobacco/ or Tobacco Products/ or "Tobacco Use Cessation Devices"/ or tobacco*.mp. or Tobacco Smoking/ | 129734 |
| 27 | Alcohol Drinking/ or alcohol*.mp. | 426657 |
| 28 | binge drinking.mp. or Binge Drinking/ | 5841 |
| 29 | Drinking Behavior/ or drinking behavio*.mp. | 11429 |
| 30 | Obesity/ or Body Weight/ or body weight*.mp. | 502154 |
| 31 | Body Mass Index/ or bmi.mp. | 212868 |
| 32 | overweight.mp. or Overweight/ | 75163 |
| 33 | obese.mp. | 129907 |
| 34 | weight gain.mp. or Weight Gain/ | 76711 |
| 35 | Weight Loss/ or weight change*.mp. | 46063 |
| 36 | sleep.mp. or Sleep/ | 195925 |
| 37 | Rest/ | 18106 |
| 38 | 15 or 16 or 17 or 18 or 19 or 20 or 21 or 22 or 23 or 24 or 25 or 26 or 27 or 28 or 29 or 30 or 31 or 32 or 33 or 34 or 35 or 36 or 37 | 3870629 |
| 39 | primary prevention.mp. or Primary Prevention/ | 33511 |
| 40 | secondary prevention.mp. or Secondary Prevention/ | 35998 |
| 41 | health promotion.mp. or Health Promotion/ | 92150 |
| 42 | 39 or 40 or 41 | 154373 |
| 43 | 5 and 9 and 14 and 38 and 42 | 823 |
| 44 | animals/ not humans/ | 4698473 |
| 45 | 43 not 44 | 814 |
| 46 | limit 45 to English language | 746 |
| *47* | *limit publication date September 2020 onwards** | *233* |

*The search was updated in January 2022, with a limitation date set on the dataset. In the original search, there were no date limitations.

**Supplementary Table 2. Individual study characteristics of 35 randomised controlled trials.**

| **Study and name (if applicable)** | **Study participants** | | | | | **Setting** | **Study arms** | **Intervention length** | **Data collection time points/follow up period** | **Retention rate** | **Study outcomes** | | | | | |
| --- | --- | --- | --- | --- | --- | --- | --- | --- | --- | --- | --- | --- | --- | --- | --- | --- |
|  | **Number** | **Age (yrs)** | **Other** | **Inclusion criteria** | **Exclusion criteria** |  |  |  |  |  | **CVD mortality/ morbidity outcome** | **Results** | **CVD risk markers** | **Results** | **Lifestyle risk factors** | **Results** |
|  | ***Primary prevention studies*** | | | | | | | | | | | | | | | |
| Chee 2020 | 26 | 40-60 | Chinese or Korean women living in the USA | Chinese or Korean (self-identified) women living in the USA; could speak English, Mandarin Chinese, or Korean. | NR | Online  USA | 1. Online program with coaching through online forums and chat groups (PA) 2. Control group with access to information on physical activity from CDC.^+^ | 3 months | 0, 1 and 3 months | 100% | None | NA | None | NA | PA: improvements in lifestyle PA (occupational activities) and planned exercise, measured on 5-point scale.* | NS |
| Folta 2009  Strong Women, Healthy Hearts | 96 | ≥ 40 | Sedentary and BMI > 24kg/m^2^ | Sedentary. Living alone | Unstable medical condition. Current participation in another lifestyle modification program. Inability to prepare food.  Cognitive impairment. Pregnant. | Face to face group sessions  USA | 1. Social cognitive therapy and behavioural strategies to improve CVD risk factors (D, PA) 2. No intervention group | 3 months | 0 and 3 months  BMI again at 3 years | 3 months: group 1: 90%, group 2: 86% | None | NA | None | NA | Diet: S/R*  PA: 2km walking test and pedometer.*  BMI and WC* | Diet: 1 ↑ vs 2  PA: 1 ↑ vs 2  BMI: 1 ↑ vs 2  WC: 1 ↑ vs 2 |
| Hageman 2014  DASHing Toward Health | 289 | 40-69 | Prehypertensive | Prehypertensive.  English speaking.  Answered “no” to all questions on the Physical Activity Readiness Questionnaire (PAR-Q) or medical clearance. | Taking anti-hypertensive medication or cortisone.  Participating in a cardiac rehab program.  Undergoing cancer treatment.  Unable to walk 1 mile continuously. | Face-to-face group sessions and online  USA | 1. Face-to-face sessions, phone calls web-based newsletters (D, PA). 2. Face-to-face sessions, phone calls printed newsletters (D, PA). 3. 1x 30-minute counselling with Dietitian (D, PA). | 12 months | 6, 12, 18 and 24 months | 6-month: 94%  12-month: 92%  18-month: 91%  24-month: 89% | None | NA | BP*  Blood lipids (total cholesterol, HDL, LDL, TG)^  BGLs^ | BP: 1 ↑ vs 3 | Diet: DASH diet (S/R)^  PA: 30-min/day (S/R)^  BMI, WC^ | Diet: 1 and 2 ↑ vs 3  WC: 1 and 2 ↑ vs 3 |
| Howard 2006  Women’s Health Initiative Dietary Modification Trial | 48,853 | 50-79 | High fat diet | Postmenopausal.  Consume a diet with fat intake of 32% or more of total calories. | On a low fat diet.  Special dietary needs.  ≥10 meals per week outside the home. Type I diabetes, colon cancer, or had any gastrointestinal conditions that contraindicated a high-fibre diet.  Bilateral prophylactic mastectomy. | Face-to-face group sessions  USA | 1. Group classes to improve D and CVD risk behaviours. 2. Dietary guidelines and other health-related pamphlets. | Unclear | 0, 12 months with 3-yearly checks | 72% | Fatal and non-fatal CVD, CHD or stroke* | NS | BP^  Total cholesterol, LDL, HDL, TG^ | 3-year BP, LDL, HDL: 1 ↑ vs 2 | BW and WC^ | BW and WC: 1 ↑ vs 2 |
| Hutchesson 2020  Be Healthe for your heart | 31 | 18-45 | Preeclampsia in last 4 years | Preeclampsia (≤4y). Internet access + email. Able to attend assessment on-site. Completed postpartum check-up at 6 weeks with no further check-up required. | Currently/recently pregnant (<3 months). Planning pregnancy within 3 months. Non-english speaking. Type 1/2 diabetes. | Online, Australia | 1. Website + email delivered (D, PA, SB + BW) 2. Wait-list control group | 3 months | 0 and 3 months | 77.4% | None | NA | BP^  Blood lipids (total cholesterol, HDL, LDL, TG)^  BGLs and insulin^ | NS | Diet: (S/R)^  PA: MET min/week, min/week of resistance training (S/R)^  SB: Weekday and weekend sitting time (S/R) ^  BW, Weight, BMI, WC, Body fat ^ | NS |
| Hwang 2020 | 22 | 18-50 | Overweight/obese | Healthy women aged between 18 to 50 years, not currently on a diet, and with BMI of 29.0–39.9 kg/m2 | CVD, hypertension or other chronic disease. Use of a antihypertensive drug.  History of tobacco use (past 6 months), currently abusing alcohol or illicit drugs. Eating disorder or use of diet pills, antioxidant pills or weight loss surgery. Pregnancy or intend to become pregnant nursing, amenorrhea. | Face-to-face and online  USA | 1. Low carb diet and caloric restriction (D). 2. Low carb diet, without caloric restriction. | 6 weeks | 0, 2, 4 and 6 weeks after intervention. | 95% | None | NA | BP^  Blood lipids (total cholesterol, HDL, LDL, TG)^  BGLs and insulin^ | NS | BMI^ | NS |
| Keyserling 2008  WISEWOMAN North Carolina | 236 | 40-64 | NA | Income below 200% of the poverty level and little or no health insurance. Participating in the National Breast and Cervical cancer early detection program. | Pregnancy/lactation. Severe chronic medical condition. | Face-to-face group sessions  USA | 1. Counselling intervention improving overall health and CVD risk (D, PA). 2. D and PA pamphlets from American Heart Association. | 12 months | 0, 6 and 12 months | 6 months: 92%  12 months: 90% | None | NA | BP^  Total and HDL cholesterol^  BGLs^ | NS | Diet: S/R*  PA: accelerometer and S/R.*  BMI^ | Diet: 1 ↑ vs 2  PA: 1 ↑ vs 2 |
| Khare 2012  WISEWOMAN Illinois | 833 | 40-64 | NA | Participating in the National Breast and Cervical cancer early detection program. | NR | Face-to-face group sessions  USA | 1. Group education (D, PA). 2. CVD risk factor screening and handouts (D, PA, BP, cholesterol, Diabetes) | 3 months | 0, 12 and 24 months | 39% | None | NA | BP*  Blood lipids (total cholesterol, LDL)*  BGLs* | NS | Diet: (S/R)*  PA: community health activities model for seniors (CHAMPS) (S/R)*  BMI* | Diet: 1 ↑ vs 2  PA: 1 ↑ vs 2 |
| Khare 2014  WISEWOMAN Illinois | 188 | 40-64 | Spanish-speaking immigrants | Participating in the National Breast and Cervical cancer early detection program. | NR | Face-to-face group sessions  USA | 1. Group education (D, PA). 2. CVD risk factor screening and handouts (D, PA, BP, cholesterol, Diabetes) | 3 months | 0, 12 and 24 months | 67% | None | NA | BP*  Blood lipids (total cholesterol, LDL)*  BGLs* | Total cholesterol and LDL: 2 ↑ vs 1 | Diet: (S/R)*  PA: community health activities model for seniors (CHAMPS) (S/R)*  BMI* | Diet: 1 ↑ vs 2  PA: 1 ↑ vs 2 |
| Kuller 2007  Women on the Move through Activity and Nutrition (WOMAN) | 508 | 52-62 | 3+ years postmenopausal, taking HRT. | Postmenopausal.  WC >80cm.  LDL100-160  BMI 25-39.9.  BP < 160/95. Beck depression score <20. 400-meter corridor walk test. | Current use of cholesterol lowering medication, dx Diabetes or on Diabetes medication. | Face-to-face group sessions  USA | 1. In-person education visits (BMI, WC, D, PA) 2. 6 seminars (D, PA) | 5-years | 0, 6 months, 12 months, 2 years, 3 years, 4 years, 5 years. | 6 months: 92.1%  18 months: 90%  48 months: 90% | None | NA | BP^  Blood lipids (HDL, LDL)^  BGLs and insulin^ | 6 months:  BP, HDL, LDL, BGLs, insulin: 1 ↑ vs 2  18 months: LDL, BGLs, insulin: 1 ↑ vs 2.  30 months: BGLs and insulin 1 ↑ vs 2 | Diet: (S/R)^  PA: MET hr/week pedometer and S/R.^  WC and BMI^ | 18 months:  Diet 1 ↑ vs 2  PA 1 ↑ vs 2  WC and BMI 1 ↑ vs 2 |
| Lawton 2008  Women’s Lifestyle Study | 1089 | 40-74 | Less than 150 minutes of moderate intensity exercise/week | Undertaking < 150 minutes of at least moderate intensity physical activity per week. | Angina, ccongestive heart failure, arrhythmia or heart valvular disease.  Severe medical conditions Hypertension (SBP ≥ 200, or DBP ≥ 120). | Telephone and face-to-face individual counseling  New Zealand | 1. ‘Lifestyle Script with exercise recommendations tailored to each individual and delivered by an ES (PA). 2. Usual care, no intervention | 9 months | 0, 12 and 24 months | 12 months: 93%  24 months: 89% | None | NA | BP^  Blood lipids (cholesterol and HDL)^  BGLs, insulin and HbA1c^ | NS | PA: S/R* | PA: 1 ↑ vs 2 |
| Low 2015 | 62 | 40-65 | Overweight | Female, 40-65yo, more than 1 lifestyle CVD risk factor. | SBP ≥200 mm Hg, DBP ≥110 mm Hg.  blood glucose levels ≥300 mg/dL. Resting heart rate ≤40 bpm. Chest pain, unstable angina, or dizziness. | Face-to-face and online  USA | 1. CVD risk reduction classes (D, PA, BW, smoking), facilitated exercise and weekly individual MI communication via phone or email. 2. CVD risk reduction classes (D, PA, BW, smoking) and access to facilitated exercise. | 6 months | 0, 6 and 12 months | 92% | None | NA | Lipids (cholesterol, LDL, HDL, TG).*  BGLs* | NS | PA: days of exercise/week (S/R)*  BMI* | NS |
| Pazoki 2006 | 385 | 25-64 | NA | No history of CVD or any other chronic disease. | NR | Face-to-face home visits  Iran | 1. Program to improve PA and incorporate into lifestyle + brochures (D, PA, smoking, CVD risk markers) 2. Usual care, no intervention | 8 weeks | 0 and 8 weeks | 87% | None | NA | BP^  Total cholesterol^  BGLs^ | BP: 1 ↑ vs 2 | PA: minutes/day and days/week (S/R)*  BMI and WC^ | PA: 1 ↑ vs 2 |
| Perry 2007  Heart to Heart | 46 | 21-65 | NA | English speaking. Physically inactive.  Living in a rural area. | History of heart disease based on self-report. Physical condition that precluded exercise.  Pregnancy | Face-to face group walking program + phone  USA | 1. Individual counselling and group-based walking program to increase PA. 2. Brief 10-minute advice session (PA) and monthly 5-minute phone call. | 12 weeks | 0 and 12 weeks. | 88% | None | NA | None | NA | PA^ | NS |
| Rich-Edwards 2019  Heart Health 4 Moms (HH4M) | 151 | 18-45 | Preeclampsia past 5y | Preeclampsia in past 5y.  Could communicate in English or Spanish. | Pregnant. History of diabetes,  CVD or kidney disease, gastric bypass, or bowel surgery, or taking medications affecting weigh/BP. | Online  USA | 1. Online website and phone calls for lifestyle advice to prevent CVD (D, PA) 2. Control website with publicly available information. | 9 months | 0, 3 and 9 months | Group 1: 93%, Group 2: 91% | None | NA | BP^ | NS | Diet: adherence to DASH (S/R)*  PA: type, duration and frequency (S/R)*  Sedentary behaviour: S/R*  BMI* | Sedentary behaviour: 1 ↑ vs 2 |
| Seguin 2018  Strong Hearts, Healthy Communities | 194 | ≥ 40 | Sedentary and BMI ≥ 25. | Sedentary.  BMI ≥ 25. BP < 160/100 mm Hg. Heart rate 60-100 beats per minute. English-speaking | Physically active.  BMI < 25. Untreated hypertension Heart rate < 60 or > than 100 beats per minute. Cognitive impairment. | Face-to-face group sessions and civic engagement  USA | 1. Group meetings and Heart club meetings (civic engagement) to learn about and engage in D + PA for improved health. 2. Less meetings covering current D + PA guidelines. | 6 months | 0 and 6 months | 78% (all assessments). | None | NA | BP^  Total cholesterol, LDL, HDL, TG^  BGLs + HbA1c^ | Total cholesterol 1 ↑ vs 2 | Diet: S/R^  PA: MET/week (S/R) and accelerometers^  BMI* | PA (S/R): 1 ↑ vs 2  BMI: 1 ↑ vs 2 |
| Seguin-fowler 2020  Strong Hearts for New York | 182 | ≥ 40 | Sedentary and BMI ≥ 25. | BMI ≥ 25.  Sedentary (< 30 minutes exercise /day).  Living in a rural postcode. | SBP > 160 or DBP > 100.  HR < 60 or > 100.  Cognitive impairment. Currently participating or planning to participate in another health behaviour change program in the next six months. | Face-to-face group sessions  USA | 1. Group meetings and Heart club meetings (civic engagement) to learn about and engage in D + PA for improved health. 2. Delayed intervention group. | 6 months | 0, 12, 24, 36 and 48 weeks. | 6 months: 72% | None | NA | BP^  Total cholesterol^  BGLs^ | NS | Diet: simple 7 questionnaire (S/R)^  PA: S/R ^  Sedentary behaviour: S/R^  BMI* | Diet: 1 ↑ vs 2  PA: 1 ↑ vs 2  BMI: 1 ↑ vs 2 |
| Simkin-Silverman 1998  Women’s Healthy Lifestyle Project | 535 | 44-50 | NA | Less than 3 months amenorrhea in 6M prior to initial telephone interview. | HRT or surgically induced menopause.  CVD risk markers.  Taking hypertensive/psychotropic medications.  Treated for cancer in past 5 years.  Participated in a weight reduction program within the past 4 months. | Face-to-face group session  USA | 1. Group education sessions (D, PA, and weight management). 2. Assessment only, no intervention | 20 weeks | 0, 6, 18, 30, 42 and 54 months. | 97% | None | NA | BP*  Total cholesterol, HDL, LDL, TG*  BGLs* | BP  Total cholesterol, HDL, LDL, TG  BGLs: 1 ↑ vs 2 | Diet: S/R^  Alcohol intake: S/R^  PA: past weekly activity (S/R).^  Smoking: daily cigarettes (S/R).^  BMI, WC, BW* | 6 months  Diet: 1 ↑ vs 2 PA: 1 ↑ vs 2  Smoking: 1 ↑ vs 2  BW and WC: 1 ↑ vs 2 |
| Staffileno 2007 | 14 | Mean 39 | 100% African American women | Sedentary lifestyle.  Premenopausal. | CVD.  Use of antihypertensive drugs.  Smoking.  Anaemia. Pregnant. Postmenopausal.  Participating in a weight loss program. | Face-to-face Individual program  USA | 1. Home-based exercise program and individual education session (PA). 2. No intervention | 8 weeks | 0, 4 and 8 weeks. | 96% | None | NA | BP* | BP: 1 ↑ vs 2 | PA: total activity and energy expenditure (S/R)* | NS |
| Staffileno 2018  The E-health Study | 35 | 18-44 | Prehypertension, 100% African American women | African American.  Untreated prehypertension (SBP 120-139 mm Hg and/or DBP 80-89 mm Hg). | NR | Online program  USA | 1. Online E-health platform improving PA. 2. Online E-health platform improving D using DASH. | 12 weeks | 0 and 12 weeks | 74% | None | NA | BP* | NS | Diet: adherance to DASH (S/R)*  PA: daily steps (S/R)* | NS |
| Stoddard 2004  WISEWOMAN Massachusetts | 1443 | 50-64 | NA | Participating in the National Breast and Cervical cancer early detection program. | NR | Face-to-face one-on-one sessions  USA | 1. Individual counselling to improve overall health and CVD risk (D, PA). 2. Assessment of CVD risk factors and referral/short counselling. | 12 months | 0 and 12 months | 80% group 1  73% group 2 | None | NA | BP*  Total cholesterol and HDL* | NS | Diet: S/R*  PA: S/R* | PA: 1 ↑ vs 2 |
| Toobert 2005  Mediterannean Lifestyle Trial | 279 | < 75 | Postmenopausal | Type 2 diabetes. Postmenopausal. Living independently.  English speaking. Living within 30 miles of the intervention site. | Developmental disability. | Face-to-face group sessions  USA | 1. Lifestyle management CVD reduction classes with ongoing support (D, PA, smoking). 2. No intervention. | 6 months | 0 and 6 months | 88% | None | NA | None | NA | Diet: adherence to Mediterranean diet (S/R)*  PA: exercise per day (S/R).*  BW* | Diet: 1 ↑ vs 2  PA: 1 ↑ vs 2  BW: 1 ↑ vs 2 |
| Witmer 2004  Traditions of the Heart | 76 | 40-64 | NA | Live within a 50-mile radius of study site, were due for an annual Pap smear test, and had previously selected one of the four participating physicians as their primary care practitioner. | NR | Face-to-face group sessions  Alaska | 1. Lifestyle assessments and lessons taught to groups (D, PA, smoking and stress). 2. Delayed intervention group | 3 months | 0, 3 and 12 months | Group 1: 61%  Group 2: 77% | None | NA | BP*  Total cholesterol, HDL and LDL* | NS | Diet: S/R*  PA frequency: S/R*  BMI and WC* | PA: 1 ↑ vs 2 |
| Wu 2014 | 100 | 40-60 | NA | Menopausal or early post menopause.  Regular menstrual patterns when 20-30 years.  Intact uterus and at least one intact ovary. | CVD.  HRT use. Medication to control CVD risk markers. Inability to perform moderate physical activity. | Online phone calls  China | 1. Phone call interviews/counselling and booklet on CVD risk factors (D, PA) 2. Usual care, no intervention. | 12 months | 0, 3, 6 and 12 months. | 3 months: 89%  6 months: 85%  12 months: 82% | None | NA | BP*  Total cholesterol, HDL, LDL, TG*  BGLs* | 3 months  BP: 1 ↑ vs 2.  6 and 12 months  BP, TG, LDL: 1 ↑ vs 2. | BW, BMI and WC* | 3 months  BW, BMI and WC: 1 ↑ vs 2.  6 months  BW, WC: 1 ↑ vs 2. |
| ***Secondary prevention studies*** | | | | | | | | | | | | | | | | |
| Beckie 2010 | 252 | >21 | NA | MI angina or undergone coronary artery bypass graft (CABG) surgery or percutaneous coronary intervention (PCI) within past year. English speaking. | Inadequate health insurance covering.  Cognitive impairment.  Inability to ambulate.  Insertion of an automatic internal cardiac defibrillator (AICD) in the last year | Face-to-face group session  USA | 1. Traditional program certified by AACPR + individual motivational counselling (PA). 2. Usual care traditional rehabilitation program. | 12 weeks | 0, 3 and 6 months | 89% | None | NA | None | NA | PA exercise attendance and completion: S/R* | PA: 1 ↑ vs 2 |
| Cornelio 2016  SALdavel | 119 | Mean 59 | Hypertension for at least 6 months | Diagnosed for hypertension for at least six months, responsible for meal preparation and ate at least five meals per week at home. | Being treated for hypertnesion in >1 health facility. | Face-to-face group sessions and phone calls  Brazil | 1. Behaviour change sessions to reduce sodium intake. 2. Usual care + general information (D, PA). | 3 months | 0 and 3 months | 77.3% | None | NA | None | NA | Diet: total salt used (S/R)* | Diet: 1 ↑ vs 2 |
| Hayashi 2010  WISEWOMAN California, Heart of the family | 1093 | 40-64 | Blood pressure and cholesterol at risk. | Hispanic. National Breast and Cervical cancer early detection program. Lack of insurance. | Women with alert values of cholesterol or blood pressure. Pregnant/ planning a pregnancy during the study period. | Face-to-face group sessions  USA | 1. Counselling intervention on improving overall health and CVD risk (D, PA) 2. Pamphlets on high BP and cholesterol | 18 months | 0, 12 and 18 months | 79% | None | NA | BP*  Total cholesterol and HDL* | BP: 1 ↑ vs 2 | Diet: S/R*  PA: S/R*  BMI* | Diet: 1 ↑ vs 2  PA: 1 ↑ vs 2 BMI: 1 ↑ vs 2 |
| Lin 2016 | 115 | ≥ 40 | Diagnosed MetS | Speak and understand Mandarin.  Walk without assistance. | Cancer, end-stage renal disease with dialysis, psychiatric disease, neurological and musculoskeletal conditions. | Face to face with or without phone calls  Taiwan | 1. Brief lifestyle modification counselling session, educational brochure and follow-up phone calls (D, PA, MetS) 2. Brief lifestyle modification counselling session and an educational brochure. 3. Usual care, no intervention. | 12 weeks | 0 and 12 weeks | Group 1: 89%  Group 2: 84%  Group 3: 87% | None | NA | BP*  Total cholesterol, HDL, LDL, TG* | HDL: 1 and 2 ↑ vs 3 | PA: weekly time of PA (S/R)* | NS |
| Mosca 2010  Secondary Prevention Beyond Hospital Walls Intervention Trial in Women (WITTI Women) | 304 | Mean 62 | Hospitalized with CHD on attainment of ACC secondary prevention guidelines | MI, angina, prior CHD, or a cardiac revascularization procedure. | Life expectancy of < 6 months.  Pregnancy Non-English or Spanish speaking. | Face-to-face and phone  USA | 1. Education and counselling during hospitalisation and through phone calls (D, PA, smoking, BW). 2. Usual care, no intervention | 12 weeks | 0, 6 weeks and 6 months | 6 weeks: 86%  6 months: 80% | None | NA | BP*  LDL* | NS | PA: S/R*  Smoking: S/R*  BMI + WC* | NS |
| Oh 2008 | 32 | > 20 | Diagnosis of MetS | Living rurally.  Abdominal obesity ( WC >80 cm). | NR | Face-to-face group sessions  Korea | 1. Group education and fitness classes for CVD prevention with MetS (D, PA) 2. Educational booklet (D, PA, MetS) | 4 weeks | 0 and 4 weeks | 78% | None | NA | BP*  Total cholesterol, LDL, HDL, TG*  BGLs* | TG: 1 ↑ vs 2 | BMI and WC* | BMI + WC: 1 ↑ vs 2 |
| Oh 2010 | 52 | 30+ | Diagnosis of MetS | Metabolic Syndrome | NR | Face-to-face group sessions  Korea | 1. Group education and fitness classes for CVD prevention with MetS (D, PA) 2. Educational booklet (D, PA, MetS) | 6 months | 0, 6 and 12 months | 92% | None | NA | BP*  Total cholesterol, LDL, HDL, TG*  BGLs* | NS | BMI and WC* | BMI + WC: 1 ↑ vs 2 |
| Schmitz 1999 | 160 | 18-70 | At least 2 CVD risk markers and smoked ≥ 5 cigarettes per day | Daily smokers.  2 known CVD risk factors. | Major psychiatric or substance-use disorders, or other serious non-coronary medical conditions. | Face-to-face individual sessions  USA | 1. Individual therapy sessions related to smoking cessation activities. 2. Presentations, handouts, and slide shows of smoking-related health information. | 6 weeks | 0, 1, 3 and 6 months. | NR | None | NA | None | NA | Smoking abstinence: S/R* | NS |
| Taha 2016 | 46 | 45-55 | Hypertensive, sedentary and smoking | Sedentary. Non-smoking.  Non diabetic.  ≤ one antihypertensive medication.  Not taking HRT or medication.  No history of CVD, renal disease, or orthopaedic conditions. | Hypertension (SBP <140 mmHg and/or DBP <90mmHg) | Face-to-face  Eqypt | 1. HIIT training 3x/week (PA). 2. Remained sedentary. | 10 weeks | 0 and 10 weeks | Unclear | None | NA | BP^ | NR | BMI | NS |
| Toobert 1998  PrimeTime | 28 | Mean 63 | Postmenopausal with CHD | Postmenopausal. Documented CHD. | Other life-threatening illnesses, MI during preceding 6 weeks, received streptokinase or alteplase, scheduled for bypass surgery. | Face-to-face group sessions USA | 1. Education, exercise groups and support groups (D, PA, stress). 2. No intervention. | 21 months | 0, 4 and 12 months | 12- and 24-months 89% | Angina and chest pain^ | NS | BP*  Total cholesterol, HDL, LDL,  TG.* | NS | Diet: S/R*  PA days and time engaged in PA: S/R*  BMI*  Smoking* | 4 and 12 months Diet + PA: 1 ↑ vs 2 |
| Tsai 2019 | 35 | 40-64 | NA | Coronary artery disease.  No history of severe arrhythmia.  Not planning to receive surgery. | Severe disease, infection, injury, or surgery.  Did not live in Taipei after hospital discharge.  Physical activity compromised. | Face-to-face individual counselling and phone calls  Taiwan | 1. Tailored lifestyle management program increasing healthy behaviours (D/PA). 2. Usual care, no intervention. | 12 weeks | 0 and 12 weeks | 94% | None | NR | BP*  Total cholesterol, HDL, LDL, TG*  BGLs* | Total cholesterol and HDL: 1 ↑ vs 2 | BMI and WC* | WC: 1 ↑ vs 2 |

All studies in this review are randomised controlled trials (RCTs).

CDC: centre for disease-control. NA: Not applicable, NS: not significant, NR: not reported

CVD: cardiovascular risk, BP: blood pressure, LDL: low-density lipoprotein, HDL: high-density lipoprotein, TG: triglycerides, BGLs: blood glucose levels, CHD: coronary heart disease. MetS: Metabolic Syndrome, MI: myocardial infarction, ES: exercise specialist, ACC: American College of Cardiology.

D: diet/nutrition, PA: physical activity, BMI: body mass index, BW: body weight, WC: waist circumference.

DASH diet: dietary approaches to stop hypertension, HRT: hormonal replacement therapy

S/R: self-report, ↑: statistically significant improvement in the respective outcome.

Only significant results reported for outcomes between groups by timepoint, if result is not reported it was not significant.

*Primary outcomes, ^Secondary outcomes.

Studies included in the meta-analysis: Folta 2009, Hageman 2014, Hayashi 2010, Howard 2006, Huang 2020, Hutchesson 2020, Keyserling 2008, Khare 2012, Khare 2014, Lawton 2008, Oh 2008, Oh 2010, Pazoki 2006, Seguin 2018, Seguin-Fowler 2020, Simkin-Silverman 1998, Staffileno 2007, Taha 2016, Toobert 1998, Tsai 2019, Wu 2014.

**Supplementary Table 3: GRADE Blood Pressure**

| **Summary of findings:** | | | | | | |
| --- | --- | --- | --- | --- | --- | --- |
| **Lifestyle interventions compared to Control in Blood Pressure for Primary and Secondary Cardiovascular Disease Prevention in Women** | | | | | | |
| **Patient or population:** Adult women ≥ 18 years old, without cardiovascular disease.  **Setting:** Face-to-face, online or hybrid interventions.  **Intervention:** Lifestyle interventions including one or more of dietary intake, physical activity, sedentary behaviour, alcohol intake, sleep quality and/or weight management.  **Comparison:** No intervention control or usual care. | | | | | | |
| Outcome № of participants (studies) | Relative effect (95% CI) | **Anticipated absolute effects (95% CI)** | | | Certainty | What happens |
|  |  |  |  | **Difference** |  |  |
| Systolic Blood Pressure (SBP) follow-up: range 1 months to 6 months № of participants: 2380 (19 RCTs) | - |  | - | MD **3.51 mmHg higher** (1.57 higher to 5.46 higher) | ⨁⨁⨁◯ Moderate^a^ | Lifestyle interventions likely results in a decrease in systolic Blood Pressure at <6 months. |
| Systolic Blood Pressure (SBP 12 Months) follow-up: range >6 months to 12 months № of participants: 3164 (10 RCTs) | - |  | - | MD **0.9 mm Hg higher** (1.36 lower to 3.15 higher) | ⨁⨁◯◯ Low^a,b^ | The evidence suggests that lifestyle interventions results in little to no difference in systolic Blood Pressure at 6-12 months. |
| Systolic Blood Pressure (SBP >12 months) follow-up: range >12 months to 36 months № of participants: 46185 (5 RCTs) | - |  | - | MD **0.95 mm Hg higher** (1.83 lower to 3.73 higher) | ⨁⨁◯◯ Low^a,b^ | The evidence suggests that lifestyle interventions results in little to no difference in systolic Blood Pressure at >12 months. |
| Diastolic Blood Pressure (DBP 6 months) follow-up: range 1 months to 6 months № of participants: 2380 (19 RCTs) | - |  | - | MD **2.25 mmHg higher** (1.68 lower to 0.84 higher) | ⨁⨁◯◯ Low^a,b^ | The evidence suggests that lifestyle interventions results in little to no difference in diastolic Blood Pressure at <6 months. |
| Diastolic Blood Pressure (DBP 12 months) follow-up: range >6 months to 12 months № of participants: 3164 (10 RCTs) | - |  | - | MD **0.33 mm Hg lower** (1.55 lower to 0.89 higher) | ⨁⨁◯◯ Low^a,b^ | The evidence suggests that lifestyle interventions results in little to no difference in diastolic Blood Pressure at 6-12 months. |
| Diastolic Blood Pressure (DBP 12 months) follow-up: range >12 months to 36 months № of participants: 46185 (5 RCTs) | - |  | - | MD **0.92 mm Hg lower** (2.41 lower to 0.57 higher) | ⨁⨁⨁◯ Moderate^b^ | Lifestyle interventions probably results in little to no difference in diastolic Blood Pressure at >12 months. |
| ***The risk in the intervention group** (and its 95% confidence interval) is based on the assumed risk in the comparison group and the **relative effect** of the intervention (and its 95% CI).  **CI:** confidence interval; **MD:** mean difference | | | | | | |
| **GRADE Working Group grades of evidence** **High certainty:** we are very confident that the true effect lies close to that of the estimate of the effect. **Moderate certainty:** we are moderately confident in the effect estimate: the true effect is likely to be close to the estimate of the effect, but there is a possibility that it is substantially different. **Low certainty:** our confidence in the effect estimate is limited: the true effect may be substantially different from the estimate of the effect. **Very low certainty:** we have very little confidence in the effect estimate: the true effect is likely to be substantially different from the estimate of effect. | | | | | | |

#### **Explanations**

a. There was a wide variance of point estimates across studies and minimal overlap of confidence intervals (CI) as seen in a forest plot.

b. Optimal information size criterion is met and there is a large sample size, however the 95% CI overlaps no effect.

**Supplementary Table 4: GRADE Total Cholesterol and High-Density Lipoprotein Cholesterol**

| **Summary of findings:** | | | | | | |
| --- | --- | --- | --- | --- | --- | --- |
| **Lifestyle interventions compared to Control in Total Cholesterol and High-Density Lipoproteins for Primary and Secondary Cardiovascular Disease Prevention in Women** | | | | | | |
| **Patient or population:** Adult women ≥ 18 years old, without cardiovascular disease.  **Setting:** Face-to-face, online or hybrid interventions.  **Intervention:** Lifestyle interventions including one or more of dietary intake, physical activity, sedentary behaviour, alcohol intake, sleep quality and/or weight management.  **Comparison:** No intervention control or usual care | | | | | | |
| Outcome № of participants (studies) | Relative effect (95% CI) | **Anticipated absolute effects (95% CI)** | | | Certainty | What happens |
|  |  |  |  | **Difference** |  |  |
| Total Cholesterol (TC 6 months) follow-up: range 1 months to 6 months № of participants: 2237 (13 RCTs) | - |  | - | MD **3.11 mg/dL higher** (1.68 lower to 7.9 higher) | ⨁⨁◯◯ Low^a,b^ | The evidence suggests that lifestyle interventions results in little to no difference in total Cholesterol at <6 months. |
| Total Cholesterol (TC 12 months) follow-up: range >6 months to 12 months № of participants: 3118 (9 RCTs) | - |  | - | MD **0.72 mg/dL lower** (5.65 lower to 4.21 higher) | ⨁⨁◯◯ Low^a,b^ | The evidence suggests that lifestyle interventions results in little to no difference in total Cholesterol at 6-12 months. |
| Total Cholesterol (TC >12 months) follow-up: range >12 months to 36 months № of participants: 46185 (5 RCTs) | - |  | - | MD **0.71 mg/dL higher** (4.81 lower to 6.24 higher) | ⨁⨁⨁◯ Moderate^b^ | Lifestyle interventions probably results in little to no difference in total Cholesterol at >12 months. |
| High Density Lipoprotein - Cholesterol (HDL-C 6 months) follow-up: range 1 months to 6 months № of participants: 1257 (13 RCTs) | - |  | - | MD **0.31 mg/dL lower** (1.68 lower to 1.06 higher) | ⨁◯◯◯ Very low^a,c^ | The evidence is very uncertain about the effect of lifestyle interventions on high Density Lipoprotein - Cholesterol at <6 months. |
| High Density Lipoprotein - Cholesterol (HDL-C >6 months) follow-up: range >6 months to 12 months № of participants: 2594 (8 RCTs) | - |  | - | MD **0.29 mg/dL higher** (1.17 lower to 1.74 higher) | ⨁◯◯◯ Very low^a,c^ | The evidence is very uncertain about the effect of lifestyle interventions on high Density Lipoprotein - Cholesterol at 6-12 months. |
| High Density Lipoprotein - Cholesterol (HDL-C >12 months) follow-up: range >12 months to 36 months № of participants: 46185 (5 RCTs) | - |  | - | MD **0.78 mg/dL higher** (0.88 lower to 2.44 higher) | ⨁⨁⨁◯ Moderate^b^ | Lifestyle interventions probably results in little to no difference in high Density Lipoprotein - Cholesterol at >12 months. |
| ***The risk in the intervention group** (and its 95% confidence interval) is based on the assumed risk in the comparison group and the **relative effect** of the intervention (and its 95% CI).  **CI:** confidence interval; **MD:** mean difference | | | | | | |
| **GRADE Working Group grades of evidence** **High certainty:** we are very confident that the true effect lies close to that of the estimate of the effect. **Moderate certainty:** we are moderately confident in the effect estimate: the true effect is likely to be close to the estimate of the effect, but there is a possibility that it is substantially different. **Low certainty:** our confidence in the effect estimate is limited: the true effect may be substantially different from the estimate of the effect. **Very low certainty:** we have very little confidence in the effect estimate: the true effect is likely to be substantially different from the estimate of effect. | | | | | | |

#### **Explanations**

a. There was a wide variance of point estimates across studies and minimal overlap of confidence intervals (CI) as seen in a forest plot.

b. Optimal information size criterion is met and there is a large sample size, however the 95% CI overlaps no effect.

c. Optimal information size criterion was not met, there is a small sample size and the 95% CI overlaps no effect.

**Supplementary Table 5: GRADE Low-Density Lipoprotein Cholesterol and Triglycerides**

| **Summary of findings:** | | | | | | |
| --- | --- | --- | --- | --- | --- | --- |
| **Lifestyle interventions compared to Control in Low-Density Lipoproteins and Triglycerides for Primary and Secondary Cardiovascular Disease Prevention in Women** | | | | | | |
| **Patient or population:** Adult women ≥ 18 years old, without cardiovascular disease.  **Setting:** Face-to-face, online or hybrid interventions  **Intervention:** Lifestyle interventions including one or more of dietary intake, physical activity, sedentary behaviour, alcohol intake, sleep quality and/or weight management.  **Comparison:** No intervention control or usual care | | | | | | |
| Outcome № of participants (studies) | Relative effect (95% CI) | **Anticipated absolute effects (95% CI)** | | | Certainty | What happens |
|  |  |  |  | **Difference** |  |  |
| Low-Density Lipoprotein - Cholesterol (LDL-C 6 months) follow-up: range 1 months to 6 months № of participants: 1089 (13 RCTs) | - |  | - | MD **1 mg/dL higher** (3.58 lower to 5.56 higher) | ⨁◯◯◯ Very low^a,c^ | The evidence is very uncertain about the effect of lifestyle interventions on low-Density Lipoprotein - Cholesterol at <6 months. |
| Low Density Lipoprotein - Cholesterol (LDL-C 12 months) follow-up: range >6 months to 12 months № of participants: 1160 (7 RCTs) | - |  | - | MD **1.34 mg/dL lower** (6.89 lower to 4.21 higher) | ⨁◯◯◯ Very low^a,c^ | The evidence is very uncertain about the effect of lifestyle interventions on low Density Lipoprotein - Cholesterol at 6-12 months. |
| Low Density Lipoprotein - Cholesterol (LDL-C >12 months) follow-up: range >12 months to 36 months № of participants: 45144 (4 RCTs) | - |  | - | MD **1.47 mg/dL higher** (4.61 lower to 7.56 higher) | ⨁⨁⨁◯ Moderate^b^ | Lifestyle interventions probably results in little to no difference in low Density Lipoprotein - Cholesterol at >12 months. |
| Triglycerides (TG 6 months) follow-up: range 1 months to 6 months № of participants: 1376 (13 RCTs) | - |  | - | MD **5.69 mg/dL lower** (0.56 lower to 11.93 higher) | ⨁◯◯◯ Very low^a,c^ | The evidence is very uncertain about the effect of lifestyle interventions on triglycerides at <6 months. |
| Triglycerides (TG >6 months) follow-up: range >6 months to 12 months № of participants: 414 (5 RCTs) | - |  | - | MD **2.39 mg/dL higher** (0.71 lower to 11.79 higher) | ⨁◯◯◯ Very low^a,c^ | The evidence is very uncertain about the effect of lifestyle interventions on triglycerides at 6-12 months. |
| Triglycerides (TG >12 months) follow-up: range >12 months to 36 months № of participants: 45096 (4 RCTs) | - |  | - | MD **0.88 mg/dL lower** (7.41 lower to 5.66 higher) | ⨁⨁⨁◯ Moderate^b^ | Lifestyle interventions probably results in little to no difference in triglycerides at >12 months. |
| ***The risk in the intervention group** (and its 95% confidence interval) is based on the assumed risk in the comparison group and the **relative effect** of the intervention (and its 95% CI).  **CI:** confidence interval; **MD:** mean difference | | | | | | |
| **GRADE Working Group grades of evidence** **High certainty:** we are very confident that the true effect lies close to that of the estimate of the effect. **Moderate certainty:** we are moderately confident in the effect estimate: the true effect is likely to be close to the estimate of the effect, but there is a possibility that it is substantially different. **Low certainty:** our confidence in the effect estimate is limited: the true effect may be substantially different from the estimate of the effect. **Very low certainty:** we have very little confidence in the effect estimate: the true effect is likely to be substantially different from the estimate of effect. | | | | | | |

#### **Explanations**

a. There was a wide variance of point estimates across studies and minimal overlap of confidence intervals (CI) as seen in a forest plot.

b. Optimal information size criterion is met and there is a large sample size, however the 95% CI overlaps no effect.

c. Optimal information size criterion was not met, there is a small sample size and the 95% CI overlaps no effect.

**Supplementary Table 6: GRADE Blood Glucose Levels and Body Mass Index**

| **Summary of findings:** | | | | | | |
| --- | --- | --- | --- | --- | --- | --- |
| **Lifestyle interventions compared to Control in Blood Glucose Levels and Body Mass Index for Primary and Secondary Cardiovascular Disease Prevention in Women** | | | | | | |
| **Patient or population:** Adult women ≥ 18 years old, without cardiovascular disease.  **Setting:** Face-to-face, online or hybrid interventions.  **Intervention:** Lifestyle interventions including one or more of dietary intake, physical activity, sedentary behaviour, alcohol intake, sleep quality and/or weight management.  **Comparison:** No intervention control or usual care. | | | | | | |
| Outcome № of participants (studies) | Relative effect (95% CI) | **Anticipated absolute effects (95% CI)** | | | Certainty | What happens |
|  |  |  |  | **Difference** |  |  |
| Blood Glucose Levels (BGLs 6 months) follow-up: range 1 months to 6 months № of participants: 2074 (14 RCTs) | - |  | - | MD **3.15 mg/dL higher** (0.16 higher to 6.14 higher) | ⨁⨁⨁◯ Moderate^a^ | Lifestyle interventions likely results in a decrease in blood Glucose Levels at <6 months. |
| Blood Glucose Levels (BGLs >6 months) follow-up: range >6 months to 12 months № of participants: 2057 (7 RCTs) | - |  | - | MD **2.33 mg/dL higher** (1.37 lower to 6.02 higher) | ⨁⨁◯◯ Low^a,b^ | The evidence suggests that lifestyle interventions results in little to no difference in blood Glucose Levels at 6-12 months. |
| Blood Glucose Levels (BGLs >12 months) follow-up: range >12 months to 36 months № of participants: 46081 (5 RCTs) | - |  | - | MD **0.52 mg/dL higher** (3.25 lower to 4.29 higher) | ⨁⨁⨁◯ Moderate^b^ | Lifestyle interventions probably results in little to no difference in blood Glucose Levels at >12 months. |
| Body Mass Index (BMI 6 months) follow-up: range 1 months to 6 months № of participants: 2232 (18 RCTs) | - |  | - | MD **0.95 kg/m2 higher** (0.54 higher to 1.35 higher) | ⨁⨁⨁◯ Moderate^a^ | Lifestyle interventions likely results in a decrease in body Mass Index at <6 months. |
| Body Mass Index (BMI >6 months) follow-up: range >6 months to 12 months № of participants: 1865 (8 RCTs) | - |  | - | MD **0.61 kg/m2 higher** (0.06 higher to 1.16 higher) | ⨁⨁◯◯ Low^a,c^ | Lifestyle interventions may result in a decrease in body Mass Index at 6-12 months. |
| Body Mass Index (BMI >12 months) follow-up: range >12 months to 36 months № of participants: 45096 (4 RCTs) | - |  | - | MD **0.58 kg/m2 higher** (0.01 higher to 1.56 higher) | ⨁⨁⨁⨁ High | Lifestyle interventions results in a decrease in body Mass Index at >12 months. |
| ***The risk in the intervention group** (and its 95% confidence interval) is based on the assumed risk in the comparison group and the **relative effect** of the intervention (and its 95% CI).  **CI:** confidence interval; **MD:** mean difference | | | | | | |
| **GRADE Working Group grades of evidence** **High certainty:** we are very confident that the true effect lies close to that of the estimate of the effect. **Moderate certainty:** we are moderately confident in the effect estimate: the true effect is likely to be close to the estimate of the effect, but there is a possibility that it is substantially different. **Low certainty:** our confidence in the effect estimate is limited: the true effect may be substantially different from the estimate of the effect. **Very low certainty:** we have very little confidence in the effect estimate: the true effect is likely to be substantially different from the estimate of effect. | | | | | | |

#### **Explanations**

a. There was a wide variance of point estimates across studies and minimal overlap of confidence intervals (CI) as seen in a forest plot.

b. Optimal information size criterion is met and there is a large sample size, however the 95% CI overlaps no effect.

c. The optimal information size criterion is not met and there is a low sample size, however the 95% CI excludes no effect

** Supplementary Figure 1: Diagnostic tools for BGLs**

**Supplementary figure 2: Mean difference time BGLs**

**Supplementary figure 3: Forrest plot BGLs**

**Supplementary Figure 4: Diagnostic tools for BMI**

**Supplementary figure 5: Mean difference time BMI**

**Supplementary figure 6: Forrest plot BMI**

**Supplementary Figure 7: Diagnostic tools DBP**

**Supplementary figure 8: Mean difference time DBP**

**Supplementary figure 9: Forrest plot DBP**

**Supplementary figure 10: Diagnostic tools HDL-C**

**Supplementary figure 11: Mean difference time HDL-C**

**Supplementary figure 12: Forrest plot HDL-C**

**Supplementary figure 13: diagnostic tools LDL-C**

**Supplementary figure 14: Mean difference time LDL-C**

**Supplementary figure 15: Forrest plot LDL-C**

**Supplementary figure 16: Diagnostic tools SBP**

**Supplementary figure 17: Mean difference time SBP**

**Supplementary figure 18: Forrest plot SBP**

**Supplementary figure 19: Diagnostic tools TC**

**Supplementary figure 20: Mean difference time TC**

**Supplementary figure 21: Forrest plot TC**

**Supplementary figure 22: Diagnostic tools TG**

**Supplementary figure 23: Mean difference time TG**

**Supplementary figure 24: Forrest plot TG**
